# Seismic evidence for a possible deep crustal hot zone beneath Southwest Washington

**DOI:** 10.1038/s41598-017-07123-w

**Published:** 2017-08-07

**Authors:** Ashton F. Flinders, Yang Shen

**Affiliations:** 1 University of Rhode Island, Graduate School of Oceanography, Narragansett, Rhode Island 02882 USA; 2 U.S. Geological Survey, California Volcano Observatory, Menlo Park, California, 94025 USA

## Abstract

Crustal pathways connecting deep sources of melt and the active volcanoes they supply are poorly understood. Beneath Mounts St. Helens, Adams, and Rainier these pathways connect subduction-induced ascending melts to shallow magma reservoirs. Petrogenetic modeling predicts that when these melts are emplaced as a succession of sills into the lower crust they generate deep crustal hot zones. While these zones are increasingly recognized as a primary site for silicic differentiation at a range of volcanic settings globally, imaging them remains challenging. Near Mount Rainier, ascending melt has previously been imaged ~28 km northwest of the volcano, while to the south, the volcano lies on the margin of a broad conductive region in the deep crust. Using 3D full-waveform tomography, we reveal an expansive low-velocity zone, which we interpret as a possible hot zone, linking ascending melts and shallow reservoirs. This hot zone may supply evolved magmas to Mounts St. Helens and Adams, and possibly Rainier, and could contain approximately twice the melt volume as the total eruptive products of all three volcanoes combined. Hot zones like this may be the primary reservoirs for arc volcanism, influencing compositional variations and spatial-segmentation along the entire 1100 km-long Cascades Arc.

## Introduction

**Figure 1:**
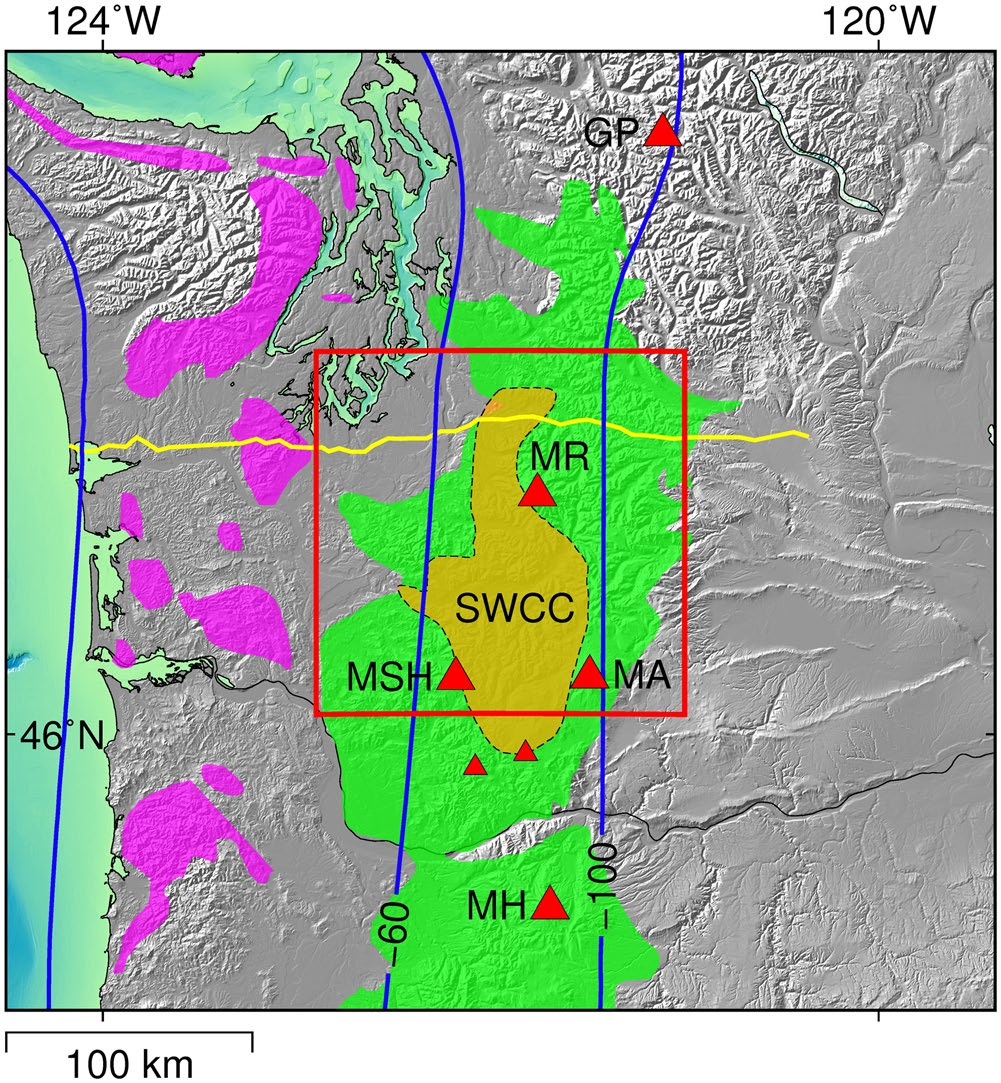
Overview of study area. Study area (red outline), depth (km) to the top of the subducting slab^47^ (blue contours), active Cascade arc volcanos (red triangles), and surface geology^45,48^. Geologic units are; Paleocene–middle Eocene submarine basalts of the Siletzia terrain (purple); chiefly Oligocene–Miocene arc igneous rocks (green). Other features; broad conductivity derived SWCC region^7^ (orange); the CAFEs MT line^8,9^ (yellow). Abbreviations given; (GP) Glacier Peak, (MR) Mount Rainier, (MSH) Mount St. Helens, (MA) Mount Adams, (MH) Mount Hood. Elevation data available from the U.S. Geological Survey. Figure made with Generic Mapping Tools^49^ (GMT) v.5.2.

Volcanism in Southwest Washington is dominated by Mounts St. Helens, Adams and Rainier, which form a triangle of recently active (<4 ka) Quaternary stratovolcanoes across the broad forearc of the Cascades^1^. Mount Rainier is one of two volcanoes in the Mounts Hood-to-Rainier segment of the arc that lie well within the forearc, 35 km west of the main Quaternary axis (Fig. 1). The other, Mount St. Helens, erupted catastrophically on May 18, 1980, resulting in 57 deaths and more than 3.2 billion USD in damages^2^ (*adjusted for inflation*). While this volcanism is ultimately driven by subduction of the Juan de Fuca Plate beneath the North American Plate, the crustal pathways connecting deep sources of melt to these volcanoes remain poorly understood. These pathways may influence the geochemical evolution of the volcanoes’ parental magmas and are thereby critical to understanding what factors help define their composition and eruptive style. At Mount Rainier, petrogenetic models propose crystallization and differentiation in multilevel reservoirs^3,4^ where small batches of magma stall as mid-crustal intrusions, often crystallizing to completion, and assimilate evolved continental sediments^3^. Subsequent injections of new magma mix with these residual silicic melts as they ascend to a shallow central reservoir^3,4^.

**Figure 2:**
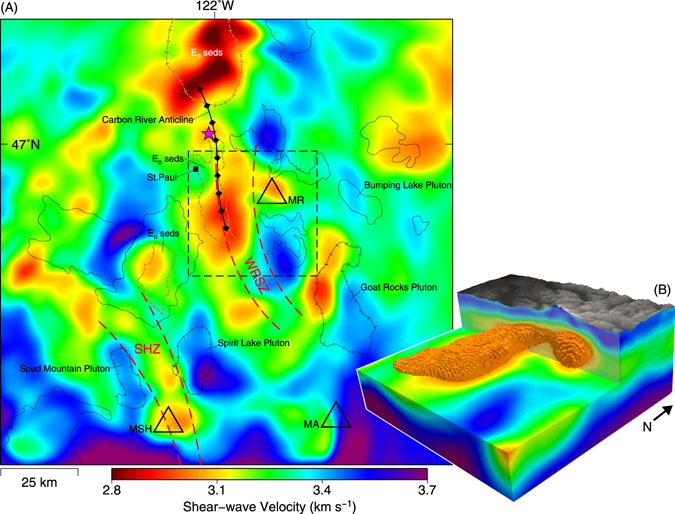
(**A**) Shear-wave velocity at 8 km depth. Fast/slow V_S_ correlate well with previously mapped lithologies. Sediment deposits^50,51^ (brown-dashed lines) are associated with slow velocities while fast velocities overlap Late Eocene to Early Miocene lava flow complexes/plutons^51–53^ (black-solid lines). Hachured lines denote the features were previously identified geophysically. Central magma reservoirs are observed beneath both Mounts Rainier and St. Helens. Approximate location of the high-conductivity reservoir imaged by McGary *et al*.^8^ shown as a purple star. Dashed rectangle outlines the 3D volume shown in (**B**). Abbreviations given; (MR) Mount Rainier, (MSH) Mount St. Helens, (MA) Mount Adams, (WRSZ) West Rainier Seismic Zone, (SHZ) Saint Helens seismic Zone, (CR) Carbon River Anticline, (St. Paul) St. Paul Lookout. (**B**) 3D Perspective of Mount Rainier’s magma system. While the imaged connection (3.05 km s^−1^ isosurface) between the WRSZ and Mount Rainier’s central reservoir is likely below our tomographic resolution, it is supported geochemically. Figure made with Generic Mapping Tools^49^ (GMT) v.5.2.

While Mount St. Helens has continued to be a target of intense tomographic imaging, Mount Rainier’s internal structure remains under studied. The volcano’s central reservoir was previously imaged as a 10-km wide slow P-wave velocity (V_P_) zone extending from 5 to 18 km depth beneath the summit of the volcano^5,6^. A second slow V_P_ zone 20 km to the west trends north-south^5^ and overlaps with a linear aeromagnetic low^7^, a region of increased shallow seismicity^5^ (Western Rainier Seismic Zone, WRSZ, Fig. 2A), and a large regional anticline^7^ (Carbon River; CR, Fig. 2A). These features within the WRSZ have been attributed to a sedimentary basement underlying the anticline, partially exposed in local Eocene sandstone and shale outcrops^5,7^, and unrelated to the volcano’s magmatic system. At the northern end of this zone, an east-west magnetotelluric (MT) profile imaged ascending fluid/melt from the top of the subducting slab to a 10 km deep, low-resistivity reservoir, ~28 km northwest of the volcano^8,9^ (purple star, Fig. 2A). While McGary *et al*.^8^ argued this reservoir provided melt to Mount Rainier, it is unclear that the MT-derived interpretations can be extended the necessary distance southeast to the volcano—particularly as their results show a resistive body at the volcano’s projected location^8,9^.

To the south and west of Mount Rainier, widely spaced MT profiles broadly outline another low-resistivity anomaly, the Southern Washington Cascades Conductor^7^ (SWCC), between Mounts St. Helens, Adams and Rainier. This anomaly has previously been interpreted as a thick (>10 km) section of marine shales containing highly saline pore fluid, and extending from ~5 to 20 km below sea level^7^ (bsl). Stanley *et al*.^7^ proposed that these shales originated from accretionary-prism/forearc-basin marine sedimentary rocks which were trapped and compressed against the pre-Tertiary margin of North American during the Eocene accretion of the Siletzia terrane^10^ (45–50.5 Ma). A later 2D MT survey of the area also attributed the SWCC to shale facies, however arguing that they could have been accreted well before basalts of the Siletzia terrane were erupted^10,11^ (>56 Ma). In this model, the shales likely originate from a section of the early Cenozoic subduction zone where an anomalously thick section of sediments had been scraped off the down going slab^11^.

An alternative and controversial interpretation of the SWCC is that it has a magmatic origin. Stanley *et al*.^7^ argued that although zones of partial melt could contribute to the low resistivity, no evidence existed for major magma bodies in the area, arguing the then recent 1980 eruption of Mount St. Helens was triggered by a very local magmatic source^12^. Egbert and Booker (1993) again did not explore a possible magmatic source for the low-resistivity, briefly noting the lack of earthquakes and surface volcanism within the interior of the SWCC. However, a more recent high-resolution MT profile between Mounts St. Helens and Adams imaged a mid-crustal anomaly extending upwards from the general vicinity and depth of the SWCC to shallow reservoirs beneath both volcanoes^13^. Contrary to previous interpretations, Hill *et al*.^13^ used the spatial correlation of this anomaly to earthquake hypocenters beneath Mount St. Helens during the 1980 eruption, and evidence for high-temperature basaltic melts residing in the lower portions of the volcano’s magma reservoir^14^, to argue the marine-sediment hypothesis for the southern extent of the SWCC was unsatisfactory. They proposed that the SWCC represents a large region of evolved partial melt supplying Mounts St. Helens and Adams.

While a lack of current surface volcanism, geothermal activity, and regions of high-heat flow within the SWCC are used as refutations to a possible magmatic origin^7,11,15^, these arguments are incomplete and exclude the possibility of lateral magmatic movements in the deep crust. Yet lateral movement of melt from deep sources has been observed at other locations^16,17^ as well as within a possibly similar deep crustal silicic body, the Altiplano-Puna “magma body” of the Central Andes^18^. Additionally, the now eroded Goat Rocks stratovolcano complex (~1 Ma) and its rhyolitic precursor the Devils Horns complex (~3.2 Ma) were long lived centers of volcanism along the eastern boundaries of the SWCC^1^. Furthermore, > 100 Quaternary volcanic vents are mapped above and in the close vicinity of the SWCC^1^ (Fig. 4). A partial-melt system the size of the SWCC would likely require hundreds of thousands to millions of years to be emplaced^19^, contemporaneous with the Goat Rocks volcano. Similarly, the main body of the SWCC, between Mounts St. Helens, Adams, and Rainier, is a regional center for high heat-flow in Southern Washington^20^. Given these considerations, along with results from the imaging Magma Under St. Helens (iMUSH) project which suggest the melt source region for the volcano lies east towards Mount Adams^21^, a magmatic origin for the SWCC should be reexamined.

Uncertainties in the crustal pathway of deep-ascending melts and their relationship to Mount Rainier’s magmatic system remain. Does the volcano’s central reservoir tap a deep crustal reservoir directly beneath the volcano (e.g. petrogenetic model^3^), the magnetotelluric-imaged northwestern reservoir (i.e. ascending melt directly from the subducting slab), or perhaps a possible SWCC-related magmatic source, as Mounts St. Helens and Adams may? Similarly, what is the origin of Southern Washington Cascades Conductor? Is it an expression of Eocene or older tectonically accreted marine sediments, or could it represent a deep magmatic source, responsible for driving the silicic differentiation observed at nearby volcanoes? If the SWCC is magmatic, is there a relationship between the melt pathways from it and the magma compositions at these three volcanoes? To address these questions, we solve for the crustal shear-wave velocity (V_S_) structure of the Mount Rainier area using a 3D full-waveform tomographic method^22,23^ and all available ambient noise data for the region (*see Data and Method*). We account for phenomena rarely considered but increasingly important at this scale and wave periods (2–30 s), including the complex 3D spatial sensitivity of wave propagation, scattering of short-period waves by topography, and P/S wave-velocity cross-dependence^22–24^. Despite the geologic complexity of the region, we see detailed correlations between our velocity structure and previously mapped lithologies (Fig. 2A). We observe a slow V_S_ anomaly in the mid-to-deep crust (~5–27 km bsl), beneath the southern Washington Cascades, that may represent the deep crustal hot zones of previous petrogenetic models.

## Tomographic Results and Interpretation

### Mount Rainier

**Figure 3:**
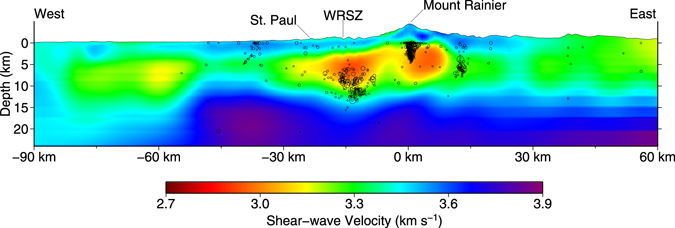
Tomographic cross-section (west-east) through Mount Rainier. Two slow V_S_ zones are associated with Mount Rainier, one under the eastern portion of the summit, the other located 10–20 km west of the volcano, coinciding with the WRSZ/Carbon River Anticline. Local earthquakes within 5 km of the profile are shown as black circles, scaled by magnitude (M1 – 4.5, from *Pacific Northwest Seismic Network* ^54^, https://pnsn.org). The western anomaly, extends deeper into the crust and is associated with deeper earthquakes. Figure made with Generic Mapping Tools^49^ (GMT) v.5.2.

**Figure 4:**
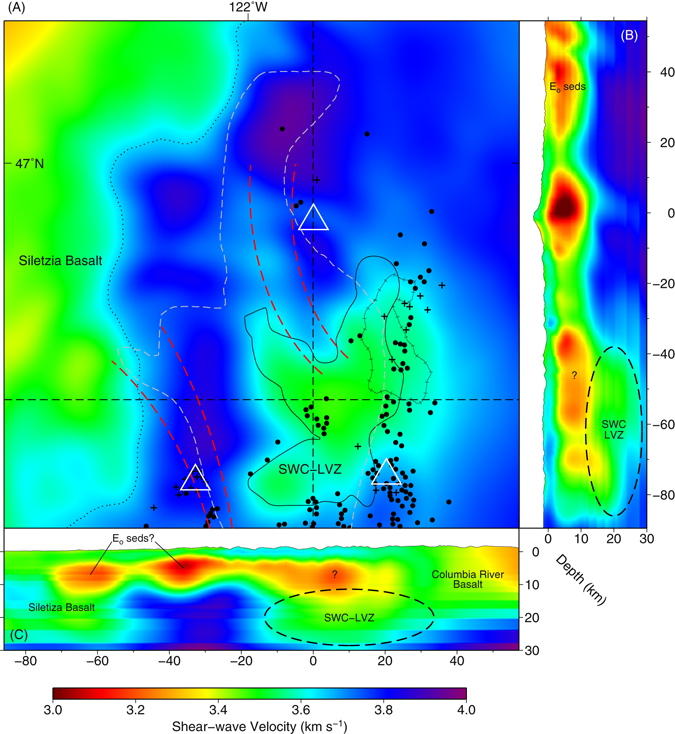
(**A**) Shear-wave velocity at 22 km depth. Submarine basalts of the Siletzia terrain occupy the western half of the model (green/reds, ~3.55 km s^−1^), until a sharp north-south divide starting ~15 km west of Mount St. Helens (dotted line). East of this divide the middle crust is dominated by pre-Eocene/Cascade arc igneous rocks and arc melange (blue/purple, ~3.75 km s^−1^), except for the slow V_S_ zone between the volcanos, the SWC-LVZ; outline of the 7% V_S_ perturbation (6% melt) shown as a solid black line. Black dots represent Quaternary volcanic vents of basaltic to andesitic composition; crosses those dacitic to rhyolitic^1^. Also shown are the SWCC from previous magnetotelluric (gray-dashed), Western Rainier Seismic Zone and Mount St. Helens Seismic Zone (red-dashed), and the Goat Rocks Pluton (hachured). Location of vertical cross-sections shown as black dashed lines. (**B**) North-south vertical cross-section. (**C**) West-east vertical cross-section. Figure made with Generic Mapping Tools^49^ (GMT) v.5.2.

Beneath Mount Rainier we observe an ~11 × 7 km WNW/ESE slow V_S_ zone (~256 km^3^, defined by a <85% V_S_ expected threshold of 3.64 ± 0.02 kms^−1^, for diorite at 5 km below the surface and ~175 °C)^25^ displaced slightly east of the summit (Figs 2A and 3, △MR). At this threshold, the zone extends from 1 to 8 km bsl (Fig. 3), with an average V_S_ of 3.0 kms^−1^, is largely aseismic (Fig. 3), and coincides with the previously observed slow V_P_ central reservoir of Moran *et al*.^5^. The 8 km basement depth agrees with the >7 km magma source depth, derived from melt inclusion volatile concentrations in andesites from the 2.2 Ka eruption^4^. Although a second mid-crustal reservoir, underneath the shallow reservoir, was proposed petrogenetically^3,4^, it is not observed in our model. Similarly, no deep-crustal magma root was observed, however a small saddle of slow velocity can be seen down to 22 km depth (Fig. 4), and these features may be too small to be resolved.

A much larger, NS trending, slow V_S_ zone is located 15–20 km west of Mount Rainier, coinciding with the WRSZ/Carbon River Anticline (Figs 2A and 3). While the contiguous portion terminates ~7 km north of the volcano, isolated anomalies extend to the shallow reservoir imaged by magnetotellurics^8,9^. Unlike beneath Mount Rainier, this slow V_S_ zone can be seen extending to depth (>15 km), is associated with deeper seismicity (Fig. 3), and possibly forms a gradual extension of a larger mid-crustal zone associated with the SWCC (Fig. 3). Additionally, an eastward prolongation of the zone may connect to the volcano’s central reservoir (Figs 2B and 3), but this lateral connection is beyond our tomographic resolution (Fig. S10).

Geochemical (major and trace elements) and isotopic (Sr, Nd, Pb, O) compositions of Mount Rainier are consistent with modest assimilation (typically ≤20 wt%) of evolved sediment^3^. This assimilation has been proposed to occur from a second mid-crustal reservoir intruding into a deep-sedimentary layer^3^. However, we find no evidence for this sedimentary layer directly beneath the volcano. Alternatively, the assimilation signature could come from direct incorporation of metasedimentary rocks (hornfels, schist, and paragneiss), or from assimilation of significantly larger volumes of Miocene intrusive rocks that had themselves assimilated sediment^3^ (Tatoosh Pluton/White River Stock). We see no high V_S_ anomalies in the shallow crust associated with intrusive rocks beneath the volcano, as we do for other plutons in the area (Figs 2A and 3).

Contrary to a deep-crustal magma reservoir beneath Mount Rainier, our tomographic model supports an alternative hypothesis in which some portion of melt ascends from the deeper crust to within the sediments of the Carbon River Anticline, undergoes crystallization and mixing, then moves laterally eastward into the central reservoir (5–8 km depth). The slow seismic velocities and aeromagnetic low of the WRSZ would then be caused by both the ascending melt, and the large Eocene sediment layer into which it intrudes. Although complex, this indirect pathway from source to surface could result from a mechanism whereby: (1) a long-lived subduction driven upwelling of melt in the lower crust exists beneath the Carbon River Anticline^8^. (2) This upwelling intrudes into a deep Eocene sediment layer, where it no longer buoyantly ascends, but creates a sill at depth. (3) The sill spreads laterally until it intrudes the preexisting network of dikes and cracks of the proto-Mount Rainier volcano^26^, active from 1–2 Mya. (4) After which, it ascends vertically to Mount Rainier’s primary magma reservoir (5–8 km). Lateral migration of magma from this off axis-western reservoir to the central reservoir is consistent with both our tomographic model and the previous petrogenic model^3^. Additionally, a long-lived magmatic source beneath the Carbon River Anticline could explain an anomalous ~2 Mya lava dome and vent (St. Paul Lookout) on the anticline’s western margin (Figs 2A and 3), ~25 km west of Mount Rainier^3^, as well a cluster of deep long-period seismic events that occur along the eastern margin of the WRSZ^5^ (Fig. 3). Similar lateral migration of melt through off-axis reservoirs has been observed at both Mount Merapi (Java) and the Kluchevskoy group (Kamchatcka) and can be attributed to similar factors^16,17^.

### Southern Washington Cascades Low Velocity Zone

In the general region of the SWCC, we image a large low velocity zone between Mounts St. Helens, Adams and Rainier, with the roof of the feature becoming noticeably deeper to the south (~15 km bsl; Fig. 4). While sensitivity testing indicates that portions of the shallow upward extension may be in part due to loss of shallow vertical resolution (Figs S11 and 12), this range of depths agree with those for the SWCC by Stanley *et al*.^7^ as well as those imaged using earthquake and controlled source seismic experiments by Parsons *et al*.^27^. We call this feature the Southern Washington Cascades Low Velocity Zone (SWC-LVZ) to distinguish it from the magnetotelluric derived boundaries of the SWCC. This distinction is important as the tomography results provide evidence that the northern extension of the previously mapped SWCC, the portion coincident with the WRSZ, is from a shallower feature, possibly unrelated to the main SWCC anomaly (Fig. 4). The northern extent of the SWC-LVZ terminates ~20–30 km south of Mount Rainier.

Limited data for shear-wave velocities of shale in the depth range of the SWC-LVZ are as low as 1.56–1.72 kms^−1^ (at 400 MPa)^28^, well below the 3.4–3.6 kms^−1^ observed (Fig. 4). Given that velocities from progressive metamorphism of shale increase with increasing metamorphic grade, and nearby Eocene-aged marine sediments range from 3.0–3.3 kms^−1^, it is likely that any Eocene-aged marine shales at these depths would have been partially metamorphosed. We can use a velocity for amphibolite-facies pelitic gneiss^29^ to assess the maximum possible contribution of these metamorphosed shales. While the mean velocity of available amphibolite-facies pelitic gneiss falls slightly above the range of the SWC-LVZ, 3.64 kms^−1^ (600 MPa and room temperature; ρ = 2.80 g/cc), the value is highly density dependent. Choosing a velocity for a pelitic gneiss with a corresponding density (2.65 g/cc) closest to the density of the SWCC (2.63 g/cc) determined by the gravity studies of Stanley *et al*.^7^, the velocity is lowered to 3.36 kms^−1^. While the shallow (<15 km) portions of the SWC-LVZ fall near this velocity, and could possibly be explained by metamorphosed shales, the deeper regions (>15 km) are significantly faster. Accounting for increased temperature at depth, any remaining pore fluid, and lower metamorphic grade would only further decrease the expected velocity, exacerbating this disparity. Shear-wave velocities for hydrated marine shales are likely too low to explain the velocities we observe for the majority of SWC-LVZ in our tomographic model.

Structurally, both the Stanley *et al*.^7^ and Wells *et al*.^10^ models for pre/Eocene accretion of marine shales imply a continuous transition from the SWCC to the basalts of the Siletzia terrane. Our tomography shows that the SWC-LVZ and the Siletzia terrane are bisected by a 20–30 km wide band of high velocity material likely associated with pre-Eocene/Cascade arc igneous rocks/mélange, pervasive in the eastern half of the model (Fig. 4A; blue/purple ~3.75 kms^−1^). Even with significant under-thrusting of the proposed accretionary-prism/forearc-basin, beneath the existing terranes to the east, it is difficult to account for this separation. Although this separation of the SWC-LVZ from the Siletzia terrane to the west does not refute the possibility of the SWCC being marine shales, it does complicate the proposed process by which the marine shales could have been accreted.

Given that more than one-hundred Quaternary volcanic vents are observed above and in the close vicinity of the SWC-LVZ (Fig. 4) and our tomographic results support neither the necessary velocities required for deep-crustal marine shales, or the mechanism by which they would have been accreted to the pre/Eocene margin, we explore the partial-melt hypothesis for the SWC-LVZ. Numerical modeling predicts that at subduction zones, when ascending hydrous-basaltic melt phase are emplaced as a succession of sills into the lower crust, they generate hot zones where mantle-derived basalt undergoes various degrees of crystallization to produce residual intermediate-to-silicic H_2_O-rich melts as well as cause partial melting of pre-existing crustal rocks^30,31^. As these hot zones evolve, gradients develop in the zone’s temperature, pressure, H_2_O content, and melt fraction, allowing for the wide range of geochemical variation seen in arc magmas^30^. Further inhomogeneity can be driven by the varying depth to the subducting slab beneath the hot zone (Fig. 1), and relative contributions of intraplate versus slab-derived components. The predominant andesitic-dacitic magmas erupted from Mounts St. Helens and Adams could have originated from the margins of this geochemical varied hot zone. This would support Hansen *et al*.’s^21^ interpretation that Mount St. Helen’s source region lies east towards Mount Adams.

More primitive basalts, such as those erupted from minor peripheral vents around Mounts Rainier and Adams, are thought to originate from conduits to deeper crustal or mantle reservoirs^1,3^. These basalts could ascend through direct pathways, within the hot zone and without undergoing sill-emplacement, or similarly through conduits on the edge of the system, bypassing it entirely. While it is evident that the northwestern extension of the SWC-LVZ underlies a portion of the WRSZ, to what extent magma intruding into the WRSZ could come directly from the mantle wedge or from the hot zone is unclear. Nevertheless, the spatial extent of the SWC-LVZ suggests it is likely one of the primary loci of melt in the region.

The proximal relationship between the SWC-LVZ and Mounts Rainier, St. Helens and Adams, and the isotopic characteristics of the volcanoes’ eruptive products are also consistent with a partial-melt hypothesis for the SWC-LVZ. Andesites and dacites from Mount St. Helens have distinctly lower K_2_O concentrations and ^206^Pb/^204^Pb ratios than those from Mounts Rainier and Adams^3^. However, small amounts of Mount St. Helens-like magmas contribute to the Mount Rainier magmatic system^3^. Mount Rainier samples selected for lower-than typical K_2_O concentrations also display low ^206^Pb/^204^Pb, and approach Mount St. Helens Nd-Sr-Pb isotope values^3^. One of several hypotheses Sisson *et al*. (2015) proposed for the origin of the low-K_2_O Mount St. Helens suite was partial melting of the buried eastern margin of the Siletzia terrane. While this shared geochemical signature could result from Mounts Rainier and St. Helens independently incorporating portions of the Siletzia terrane near the base of their magma reservoirs, it could also occur from deeper, direct incorporation, by the western margin of a SWC-LVZ partial melt zone in the mid-to-lower crust (Fig. 4). Differences in K_2_O concentrations would then reflect the local availability of the SWC-LVZ zone to incorporate Siletzia basalt and the preservation of this signature as melt moves from the SWC-LVZ to the volcano. Mount St. Helens and the southwestern edge of the SWC-LVZ lay relatively close to one another, as well as the edge of the Siletzia terrane (Fig. 4A), consistent with strong assimilation of Siletzia-influenced partial melt. Mount Rainier, further east of the Siletzia boundary and further north of the SWC-LVZ, exhibits a relatively minor but still detectable contribution, modified by incorporation of WRSZ sediment with lower ^143^Nd/^144^Nd and higher ^87^Sr/^86^Sr, ^207^Pb/^204^Pb. Mount Adams, with the eastern edge of the SWC-LVZ being far from the Siletzia boundary, shows no ^206^Pb/^204^Pb evidence for its incorporation^3^. Additionally, there is no Nd-Sr-Pb isotope evidence for crustal sediment assimilation in Mount Adam’s magmas^3^, expected if the SWC-LVZ and SWCC were partially of sedimentary origin.

## Reservoir Characteristics

### Mount Rainier and Mount St. Helens

Although Mount Rainier’s slow V_P_ central reservoir has been loosely constrained to the combined effects of heated rock and a small melt fraction^5^, with our new model we can attempt to estimate individual contributions to our observed ~15% V_S_ anomaly (0.64 kms^−1^). Oxide geothermometry gives a past pre-eruptive temperature of 980 °C for the volcano’s 2.2 ka eruption central-reservoir andesites^4^. While this temperature may overestimate the current thermal conditions, it is, in part, supported by the aseismicity of the slow V_S_ zone (Fig. 3), suggesting temperatures above the brittle-ductile transition^32^ (600-900 °C). Although no experimentally derived V_S_ temperature derivative was available for diorite, we can use the range given by granite (−0.21 × 10^−3^ kms^−1^C^−1^) and peridotite (−0.39 × 10^−3^ kms^−1^C^−1^) as end-member proxies^32^. If we assume the entire volume is held at the 980 °C pre-eruptive temperature—likely an over estimate of the thermal contribution—this excess above the 35 °C km^−1^ geothermal gradient^20^ accounts for ~38% ± 16% of the observed slow V_S_. This temperature contribution is estimated at the mean depth of the slow V_S_ zone, 3.5 km bsl, or ~ 5 km below the surface. Moran *et al*. (2000) attributed the majority of volcano-tectonic (VT) earthquakes beneath the volcano to processes associated with magmatic-hydrothermal fluid circulation, and thus the remaining anomaly can be attributed to either hydrothermal fluids, melt, or some combination of the two. Without additional constraints (such as V_P_/V_S_ ratios) we calculate the maximum melt contribution by assuming the remaining anomaly is due to melt alone. Shear-wave velocity versus crystal-fraction experiments for a synthetic crystal-liquid suspension^33,34^ with ~5 wt% H_2_O, extrapolated to the 980 °C reservoir temperature^4^, allows us to estimate that each percent of melt leads to a decrease in V_S_ of ~0.07 kms^−1^. The residual slow V_S_ (~0.40 kms^−1^ ± 0.1 kms^−1^) is accounted for by ~5% melt, or a maximum of ~13 km^3^ of melt in the 256 km^3^ imaged reservoir. While the melt-fraction to shear-wave velocity relationships used here are based on experiments collected at ultrasonic frequencies (3 MHz) in an unrelaxed regime, at small melt fractions (<10%) scaling to seismic frequencies becomes largely inconsequential^35^. For comparison, trends of V_S_ versus porosity (melt/water-CO_2_ filled) for granitic systems, compiled by Chu *et al*.^35^, estimate a V_S_ of ~3.2 kms^−1^ for a system with 5% melt. Adjusting their estimate to account for our elevated reservoir temperature yields a V_S_ of 3.0 ± 0.1 kms^−1^, in agreement with the of 3.0 kms^−1^ velocity we observed beneath Mount Rainier.

As tomography provides average V_S_ solutions over spatial scales larger than individual melt-filled dikes, sills, and fissures, it can overestimate volumes and underestimate melt-fractions. For comparison, a similar estimate (<85% V_S_ expected) for the melt fraction for the Mount St. Helens central-magma reservoir (Fig. 2A, △MSH; T_res_ = 850 °C, expected V_S_ = 3.61 kms^−1^, V_S_/T = −0.21 × 10^−3^ kms^−1^ C^−1^, ∂V_S_/∂melt-fraction = 0.07 kms^−1^/%melt) results in a melt fraction of ~9%, and a reservoir volume of 375 km^3^, or ~33 km^3^ of melt. This melt volume is on the order of estimates provided by geodetic modeling of the 2004-2006 eruptive period^36^ (10–25 km^3^), and possibly overestimated as our model does not account for bubble fraction. While melt fractions for Mounts Rainier and St. Helens are within the ranges found for other volcanoes using seismic tomography; <4% melt Taupu Volcano^37^, 10% melt Kilauea’s East Rift Zone^38^, and 3–10% melt Soufrière Hills^39^—they are, similarly, underestimates of the true maximum melt fraction, averaged over a volume larger than the true magma body. For example, at Soufrière Hills, when the tomographic melt-fraction estimates were constrained by numerical models of magma reservoir growth, significantly higher melt fractions (>30%) were found, in agreement with petrological observation^39^. The tomographic model-based assessments of melt reservoirs for both Mount Rainier and St. Helens likely underestimate melt fraction while overestimating reservoir volume, with the estimated melt volumes likely being much more robust.

### Southern Washington Cascades Low Velocity Zone

**Figure 5:**
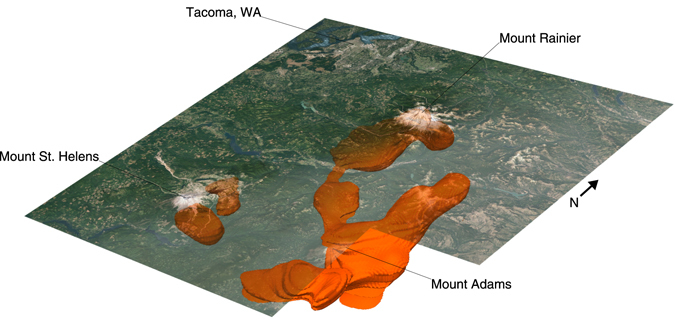
Aerial Image of Southwest Washington and 7% Slow V_s_ Isosurface (~6% melt). The SWC-LVZ defined by the >7% slow V_S_ isosurface, equivalent to ~6% partial melt (volume/melt-fractions calculations exclude the WRSZ and Mount Rainier magma reservoir). Additional slow bodies are shown for Mount Rainier’s/St. Helen’s magmatic system, the WRSZ, and the SHZ. The city of Tacoma Washington is seen to the northwest. Imagery available from the U.S. Geological Survey. Figure made with Generic Mapping Tools^49^ (GMT) v.5.2.

Although we lack the well-defined lithologic and thermal constraints available for Mounts Rainier and St. Helens, we can estimate a possible melt-fraction of the SWC-LVZ by calculating its percent perturbation from the surrounding pre-Eocene/Cascade arc igneous rocks and arc melange. We derive this expected velocity from an average of 25 depth/velocity profiles through the middle/eastern half of the model (blue areas Fig. 4), specifically excluding regions associated with the Siletzia terrane, sedimentary deposits, volcanic-reservoirs/plutons/intrusive complexes, and the SWC-LVZ itself. We subtract this average velocity profile from the original model, and threshold the result so only negative perturbations are visible (regions with slower than expected velocities). The maximum threshold for the negative perturbation is incrementally adjusted until the majority of the arc igneous crust is no longer visible, and the SWC-LVZ is a separate contiguous body (~−7% perturbation; Figs 4 and 5). This body has an estimated volume >12,800 km^3^, with an average reduced V_S_ of 0.30 ± 0.04 kms^−1^, or −8% from expected (excluding the WRSZ; Figs 4 and 5). To the south, near Mounts St. Helens and Adams it has a minimum depth of ~15 km bsl, shoaling to ~10 km bsl at its northern extent. Although likely tomographically unconstrained, it extends to ~27 km bsl. If we assume mid-crustal temperature could range from 400–800 °C^8,20,21^ each percent of interstitial melt leads to a decrease in V_S_ of ~0.05 ± 0.01 kms^−1^. The total V_S_ anomaly could then be accounted for by an average of ~6% melt, or 768 km^3^, ~2× the total eruptive volume (365 km^3^) of the Mounts St. Helens-Adams-Rainier volcanoes combined^1^. Our estimate of melt content is within the range of recent estimates for the SWCC (2–12%) based on high-pressure conductivity and petrological experiments^40^.

## Conclusion

We propose that the SWC-LVZ and SWCC could be indicative of large-scale basaltic sill emplacement and silicic differentiation^35^ in the Southern Washington Cascades and represent the primary reservoir of the region’s Quaternary arc magmatism. Compositional variations in nearby volcanoes may be, in part, controlled by their location relative to the SWC-LVZ, and the subsurface pathways of the ascending hydrous-basaltic melt phase. Mount Rainier stands on the SWC-LVZ’s diminishing northern margin, coinciding with an abrupt end of the nearly continuous Mounts Hood-to-Rainier segment of the Cascade Arc and the disappearance of Quaternary volcanic vents to the north. The next Quaternary cluster to the north (south of Glacier Peak) is separated by a 120 km-long-gap (Fig. 1), and we hypothesize that these segments/gaps may be controlled by regions of influence of SWC-LVZ-like deep crustal hot zones throughout the entire 1100 km-long Cascades Arc. Differences in volcanism between the Mounts Hood-to-Rainier Segment and the neighboring Garibaldi Volcanic Belt to the north, and Oregon Cascades Segment to the south, could be partially controlled by the size and emplacement depth of these crustal hot zones. Further detailed imaging of the crust along the length of the arc is needed to confirm the presence of these hot zones. Correlating their locations/extents to Quaternary volcanic centers/intrusions will provide valuable insight on the timescales involved for active volcanism and material cycling in subduction zones.

## Data and Method

### Empirical Green’s Functions

Source data are derived from the cross-correlation of ambient noise waveforms, a well-proven technique to reconstruct Empirical Green’s Function (EGFs) between two seismic-station pairs, i.e., the station’s far-field response to an impulsive source at the paired station. EGFs were extracted from short-period (EHZ) and broadband (BHZ) high-gain vertical-component seismic stations within 110 km of Mt. Rainier (Fig. S1; 99 stations total). Data requests were made through the Incorporated Research Institution for Seismology (IRIS) Data Management Center, and limited to the years 2000–2014. Processing included removing the seismometer instrument response, cutting the records into daily segments, normalizing their spectra, and bandpass filtering from 0.5–40 s. Normalization was performed via the frequency-time normalization method^41^ using 6.25 mHz wide frequency bands. This method normalizes the record across all frequencies, attempting to compensate for frequency-dependent amplitude loss and energy partitioning—however, amplitudes are not used in the tomographic process beyond signal-to-noise discriminations (relative). Portions of seismic records overlapping with large earthquakes (M > 5.0) were nulled and tapered. Daily records for unique station-station pair combinations were cross-correlated and stacked by month, and EGFs calculated as their time derivative. EGFs for months with signal-to-noise ratios <5 were removed from the database. This discrimination removed months where instruments were behaving erratically or when there was localized coherent noise. All daily cross-correlations for the remaining months were then stacked into a final cross-correlation record, and subsequent EGFs calculated (Fig. S2; 2607 EGFs).

### Full-Waveform 3D Simulation and Inversion

While application of ambient noise records to volcano-seismic tomography has become more popular, forward modeling in general has been limited to classical approaches (non-3D/ray-based methods). Ray-based methods assume that the propagation time of a wave from source-to-receiver is only sensitive to velocity perturbations along a line-path connecting the two locations. This assumption does not account for the 3D spatial sensitivity of wave propagation, necessary to accurately invert for a complex 3D velocity model. 3D full-waveform methods have been shown to yield improved data fits over classical non-3D/ray-based approaches^42,43^.

Our full-wave tomographic method accounts for complex wave propagation using an iterative approach. We follow the general procedures of *Gao and Shen* (2014) where each iteration is comprised of (1) finite-difference wave propagation simulations, (2) measurement of phase delays between observed (EGFs) and synthetic waveforms, (3) calculation of 3D phase-delay sensitivity kernels to V_P_ and V_S_ perturbations using the strain Green tensor scattering-integral approach^22^, (4) inversion for velocity perturbations and (5) updating the velocity model. Unlike in Gao and Shen (2014), which used a finite-difference code in the spherical coordinate system, we use a curvilinear version of the 3D nonstaggered-grid finite-difference method of Zhang *et al*.^44^ to model the propagation of short-period Rayleigh surface waves (up to 0.5 Hz) between station-station pairs, using a starting model derived from a previous regional shear-wave ambient noise tomography model^45^ (Fig. S3). A vertical force, with a Gaussian source-time function and a half width of 1 s, was applied at a station location and the resultant surface particle velocity wavefield was used to calculate synthetic Green’s functions (SGFs) for all stations paired with the source station (Fig. S4). Delay times between SGFs and EGFs were then calculated for a range of discrete overlapping frequency bands (Fig. S5). Full 3D Green tensor volumes, saved for each station simulation, were used to calculate 3D sensitivity kernels to V_P_ and V_S_ perturbations (Fig. S6). These frequency-band dependent sensitivity kernels and delay times were then used to invert for the velocity structure, using a standard LSQR routine^46^ (Figs S7 and 8) with constant regularization parameters. For computational efficiency and solution stability, the long-wavelength velocity structure of the region was solved for first. Iterations 1-8 used a 500 m xy-grid spacing and the frequency bands (4.5–9 s, 6.75–13.5 s, 10.125–20.25 s), while iterations 9–10 used a 250 m xy-grid spacing and two additional high-frequency bands (2–4 s, 3–6 s) and one lower-frequency band (15.2–30.4 s). Vertical grid spacing was constant through all iterations and graded from 200 m near the surface to 1 km at the base of the model, which extended to 90 km depth.

### Data Availability

Empirical Greens’ Functions data used in this study are available from the IRIS DMC as a compiled dataset, report number 16-024, nickname RAINIER-ANT-FWI; http://ds.iris.edu/data/reports/2016/16-024.

## Electronic supplementary material

Supplementary Figures

Tomography Results Animation Legend

Tomography Results Animation

## References

[1] Hildreth, W. Quaternary magmatism in the Cascades-geologic perspectives. *U.S. Geol. Surv. Prof. Pap*. **1744** (2007).

[2] Hunt, C. E. & MacCready, J. S. The short-term economic consequences of the Mount St. Helens volcanic eruptions in May and June, 1980, Washington State Department of Commerce and Economic Development 94 p (1980).

[3] Sisson TW, Salters VJM, Larson PB (2013). Petrogenesis of Mount Rainier andesite: Magma flux and geologic controls on the contrasting differentiation styles at stratovolcanoes of the southern Washington Cascades. Geol. Soc. Am. Bull..

[4] Venezky DY, Rutherford MJ (1997). Preeruption conditions and timing of dacite-andesite magma mixing in the 2.2 ka eruption at Mount Rainier. J. Geophys. Res..

[5] Moran SC, Lees JM, Malone SD (1999). Pwave crustal velocity structure in the greater Mount Rainier area from local earthquake tomography. J. Geophys. Res..

[6] Obrebski M, Abers GA, Foster A (2015). Magmatic arc structure around Mount Rainier, WA, from the joint inversion of receiver functions and surface wave dispersion. Geochem. Geophys. Geosyst..

[7] Stanley WD, Finn C, Plesha JL (1987). Tectonics and conductivity structures in the Southern Washington Cascades. J. Geophys. Res..

[8] McGary RS, Evans RL, Wannamaker PE, Elsenbeck J, Rondenay S (2014). Pathway from subducting slab to surface for melt and fluids beneath Mount Rainier. Nature.

[9] Wannamaker PE (2014). Segmentation of plate coupling, fate of subduction fluids, and modes of arc magmatism in Cascadia, inferred from magnetotelluric resistivity. Geochem. Geophys. Geosyst..

[10] Wells R (2014). Geologic history of Siletzia, a large igneous province in the Oregon and Washington Coast Range: correlation to the geomagnetic polarity time scale and implications for a long-lived Yellowstone hotspot. Geosphere.

[11] Egbert GD, Booker JR (1993). Imaging crustal structure in southwestern Washington with small magnetometer arrays. J. Geophys. Res..

[12] Weaver CS, Grant WC, Shemeta JE (1987). Local Crustal Extension at Mount St. Helens, Washington. J. Geophys. Res..

[13] Hill GJ (2009). Distribution of melt beneath Mount St Helens and Mount Adams inferred from magnetotelluric data. Nat. Geosci..

[14] Heiker C (1995). Inclusions in Mount St. Helens Dacite Erupted from 1980 through 1983. J. Volcanol. Geoth. Res..

[15] Sanderson, K. Supervolcano? Or just hot air? *Nature News Blog* at http://blogs.nature.com/news/2009/10/supervolcano_or_just_hot_air.html (2009).

[16] Wagner D (2007). Joint inversion of active and passive seismic data in Central Java. Geophys. J. Int..

[17] Koulakov I (2011). Feeding volcanoes of the Kluchevskoy group from the results of local earthquake tomography. Geophys. Res. Lett..

[18] Ward KM, Zandt G, Beck SL, Christensen DH, McFarlin H (2014). Seismic imaging of the magmatic underpinnings beneath the Altiplano-Puna volcanic complex from the joint inversion of surface wave dispersion and receiver funtions. Earth Planet. Sci. Lett..

[19] de Silva SL, Greg PM (2014). Thermomechanical feedbacks in magmatic systems: implications for growth, longevity, and evolution of large caldera-forming magma reservoirs and their supereruptions. J. Volcanol. Geoth. Res..

[20] Blackwell DD, Steele JL, Kelley S, Korosec MA (1990). Heat flow in the state of Washington and thermal conditions in the Cascade Range. J. Geophys. Res..

[21] Hansen, S. M. *et al*. Seismic evidence for a cold serpentinized mantle wedge beneath Mount St Helens. *Nature Comm*. **7** (2016). 10.1038/ncomms13242 PMC5097125 27802263

[22] Zhao L, Jordan TH, Olsen KB, Chen P (2005). Frechet Kernels for Imaging Regional Earth Structure Based on Three-Dimensional Reference Models. Seismol. Soc. Am. Bull..

[23] Zhang W, Shen Y (2010). Unsplit complex frequency-shifted PML implementation using auxiliary differential equations for seismic wave modeling. Geophysics.

[24] Zhang Z, Shen Y (2008). Cross-dependence of finite-frequency compressional waveforms to shear seismic wave speeds. Geophys. J. Int..

[25] Christensen NI (1996). Poisson’s ratio and crustal seismology. J. Geophys. Res..

[26] Fiske RS, Hopson CA, Waters AC (1963). Geology of Mount Rainier National Park, Washington. U.S Geo. Survey Prof. Paper.

[27] Parsons T, Wells RE, Fisher MA (1999). Three-dimensional velocity structure of Siletiza and other accreted terranes in the Cascadia forearc of Washington. J. Geophys. Res..

[28] Shaocheng, J., Wang, Q., & Xia, B. *Handbook of Seismic Properties of Minerals, Rocks, and Ores*. 1–630 (Polytechnic International Press, 2002).

[29] Rudnick RL, Fountain DM (1995). Nature and composition of the continental crust: a lower crustal perspective. Rev. Geophys..

[30] Annen C, Blundy JD, Sparks RSJ (2005). The Genesis of Intermediate and Silicic Magmas in Deep Crustal Hot Zones. J. Petrol..

[31] Solano JMS (2012). Melt Segregation in Deep Crustal Hot Zones: A Mechanism for Chemical Differentiation, Crustal Assimilation, and the Formation of Evolved Magmas. J. Petrol..

[32] Tuffen H, Smith R, Sammonds PR (2008). Evidence for seismogenic fracture of silicic magma. Nature.

[33] Kern H, Richter A (1981). Temperature derivatives of compressional and shear wave velocities in crustal and mantle rocks at 6 kbar confining pressure. J. Geophys..

[34] Caricchi L, Burlini L, Ulmer P (2008). Propagation of P and S-waves in magmas with different crystal contents: Insights into the crystallinity of magmatic reservoirs. J. Volcanol. Geotherm. Res..

[35] Chu R, Helmberger DV, Sun D, Jackson JM, Zhu L (2010). Mushy magma beneath Yellowstone. Geophys. Res. Lett..

[36] Mastin LG, Lisowski M, Roeloffs E, Beeler N (2009). Improved constraints on the estimated size and volatile content of the Mount St. Helens magma system from the 2004–2008 history of dome growth and deformation. Geophys. Res. Lett..

[37] Heise W (2007). Melt distribution beneath a young continental rift: The Taupo Volcanic Zone, New Zealand. Geophys. Res. Lett..

[38] Lin G, Amelung F, Lavallee Y, Okubo PG (2014). Seismic evidence for a crustal magma reservoir beneath the upper east rift zone of Kilauea volcano, Hawaii. Geology.

[39] Paulatto M (2012). Magma chamber properties from integrated seismic tomography and thermal modeling at Montserrat. Geochem. Geophys. Geosyst..

[40] Laumonier M, Gaillard F, Muir D, Blundy J, Unsworth U (2017). Giant magmatic water reservoirs at mid-crustal depth inferred from electrical conductivity and the growth of the continental crust. Earth Planet. Sci. Lett..

[41] Ekström G, Abers GA, Webb SC (2009). Determination of surface-wave phase velocities across USArray from noise and Aki’s spectral formulation. Geophys. Res. Lett..

[42] Maceira M, Larmat C, Porritt RW (2015). On the validation of seismic imaging methods: Finite frequency or ray theory?. Geophys. Res. Lett..

[43] Gao H, Shen Y (2014). Upper mantle structure of the Cascades from full-wave ambient noise tomography: Evidence for 3D mantle upwelling in the back-arc. Earth Planet. Sci. Lett..

[44] Zhang W, Zhang Z, Chen X (2012). Three-dimensional elastic wave numerical modelling in the presence of surface topography by a collocated-grid finite-difference method on curvilinear grids. Geophys. J. Int..

[45] Walker, G. W. & MacLeod, N. S. Geologic Map of Oregon, *U.S. Geol. Survey*, scale 1:500,000 (1991).

[46] Paige CC, Saunders MA (1982). LSQR: An algorithm for Sparse Linear Equations and Sparse Least Squares. Trans. Math. Soft..

[47] McCrory PA, Blair JL, Oppenheimer DH, Walter ASR (2006). Depth to the Juan de Fuca Slab Beneath the Cascadia Subduction Margin—A 3-D Model for Sorting Earthquakes. USGS Data Series.

[48] Schuster J. E. Geologic map of Washington State, Washington Division of Geology and Earth Resources Geologic Map GM-53, 1:500,000 (2005).

[49] Wessel, P., Smith, W. H. F., Scharroo, R., Luis, J. F. & Wobbe, F. Generic Mapping Tools: Improved version released. *EOS Trans*. **94**, 409–410. http://gmt.soest.hawaii.edu/ (2013).

[50] Lees JM, Crosson RS (1990). Tomographic imaging of local earthquake delay times for three-dimensional velocity variation in western Washington. J. Geophys. Res..

[51] Hammond PE (1998). Tertiary Andesitic Lava-flow Complexes (Stratovolcanoes) in the Southern Cascade Range of Washington–Observations on Tectonic Processes within the Cascade Arc. Washington Geo..

[52] Lees JM, Crosson RS (1989). Tomographic inversion for three-dimensional velocity structure at Mount St. Helens using earthquake data. J. Geophys. Res..

[53] Mattinson, J. M. Emplacement history of the Tatoosh volcanic-plutonic complex, Washington: Ages of zircons. *Geol. Soc. Am. Bull*. (1977).

[54] University of Washington. Pacific Northwest Seismic Network. *International Federation of Digital Seismograph Networks*. Other/Seismic Network (1963).

