# Publisher Correction: Seismic evidence for a possible deep crustal hot zone beneath Southwest Washington

**DOI:** 10.1038/s41598-017-16212-9

**Published:** 2017-11-22

**Authors:** Ashton F. Flinders, Yang Shen

**Affiliations:** 1 University of Rhode Island, Graduate School of Oceanography, Narragansett, Rhode Island 02882 USA; 2 U.S. Geological Survey, California Volcano Observatory, Menlo Park, California, 94025 USA

*Scientific Reports* **7**:7400; doi:10.1038/s41598-017-07123-w; Article published online 7 August 2017

The original PDF version of this Article contained lower resolution figures. This has now been corrected in the PDF; the HTML version of the paper was correct from the time of publication.

