# Supplementary material for: Seismic evidence for a possible deep crustal hot zone beneath Southwest Washington
Source: Sci Rep. 2017 Aug 7;7:7400. doi: 10.1038/s41598-017-07123-w (PMC5547095; doi:10.1038/s41598-017-07123-w)
Supplement: Supplementary file 1 — Supplementary Figures [file 41598_2017_7123_MOESM1_ESM.pdf]

Seismic evidence for a possible deep crustal hot zone beneath Southwest Washington

Ashton F. Flinders[1,2]*, Yang Shen[2]

[1]U.S. Geological Survey, California Volcano Observatory, Menlo Park, California, 94025
[2]University of Rhode Island, Graduate School of Oceanography, Narragansett, Rhode Island, 02882

Supplementary Materials

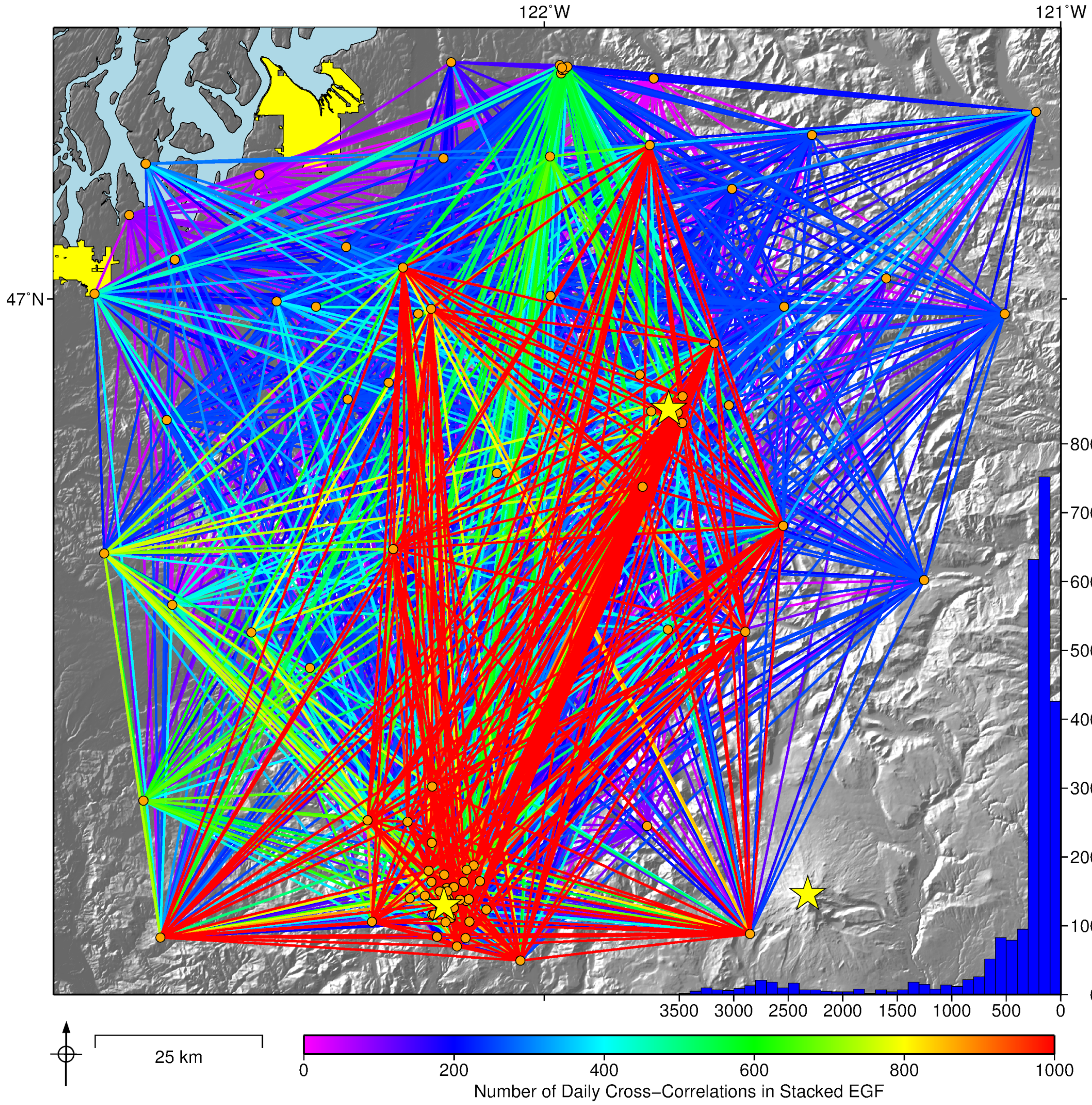

Supplemental Figure 1. Station locations, ray-paths, and stacked distributions of empirical Green's functions. Seismometer locations (orange circles) relative to volcano locations (yellow-stars). The number of daily cross-correlations in the final stacked empirical Green's function is shown by both the colored ray-path between station locations, and the inset histogram. Digital elevation map available from the U.S. Geological Survey.

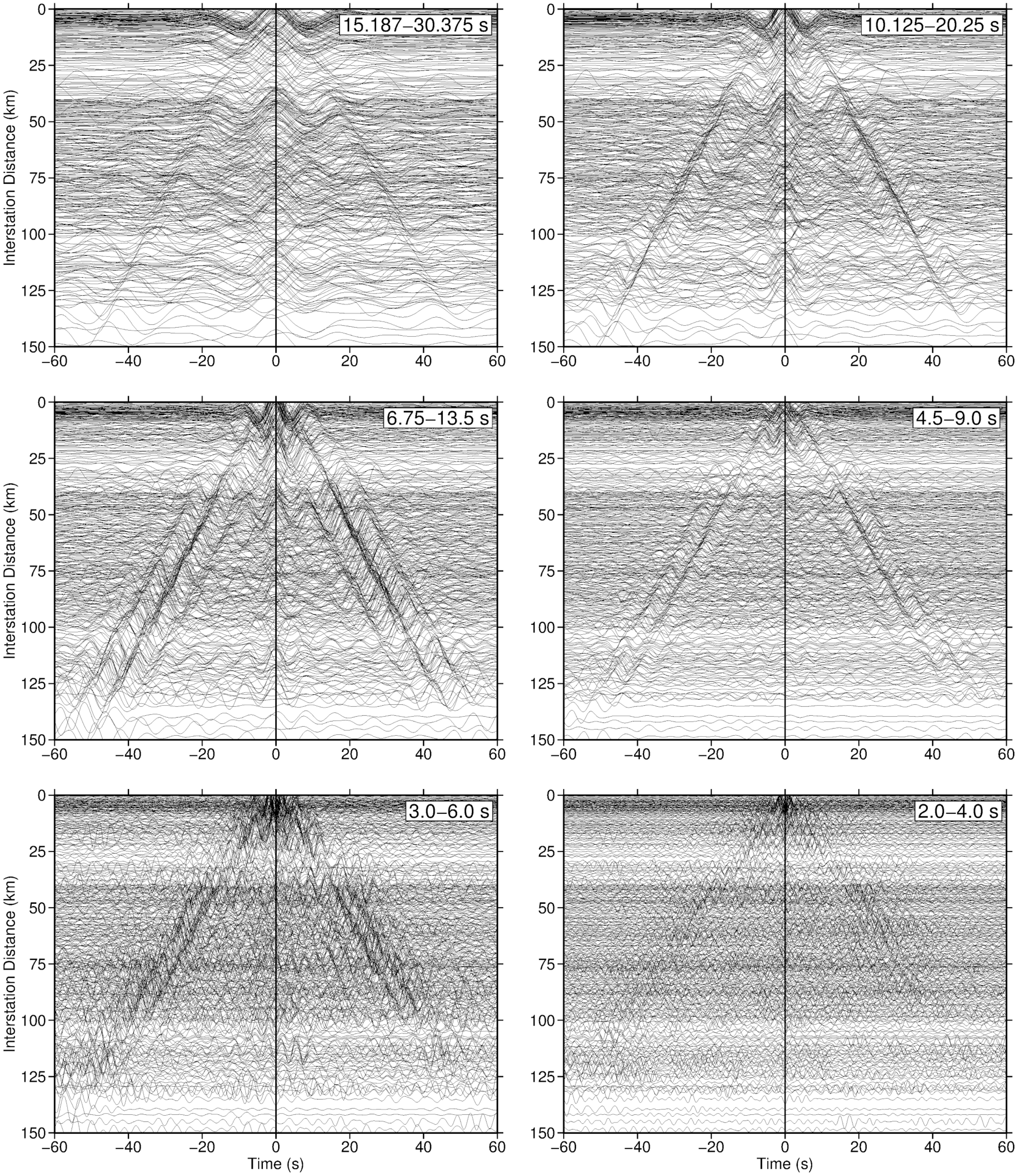

Supplemental Figure 2. Empirical Green's functions versus distance. The six different frequency bands used in the tomography, showing clear move out of the surface wave arrival.

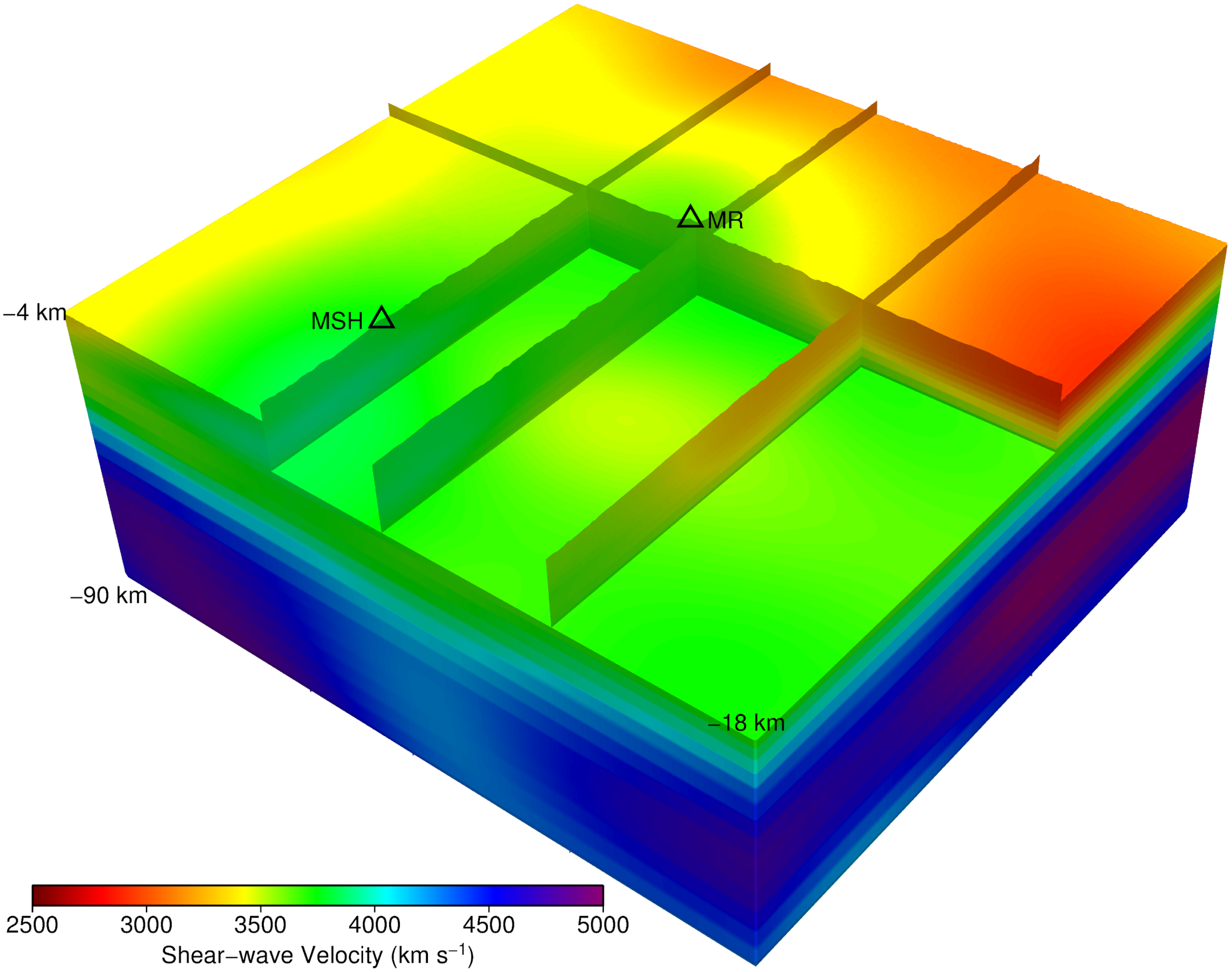

Supplemental Figure 3. 3D starting model. The starting model for tomography was taken from the regional *Gao and Shen* (2014) model. While this model resolved some deep-crustal/upper-mantle structure, there was no small-scale structure in the middle-to-upper crust. There was also no noticeable change in the velocity structure from east-to-west characteristic of the border of the Siletzia terrane. A small deep crustal low velocity anomaly may be observable at 18 km.

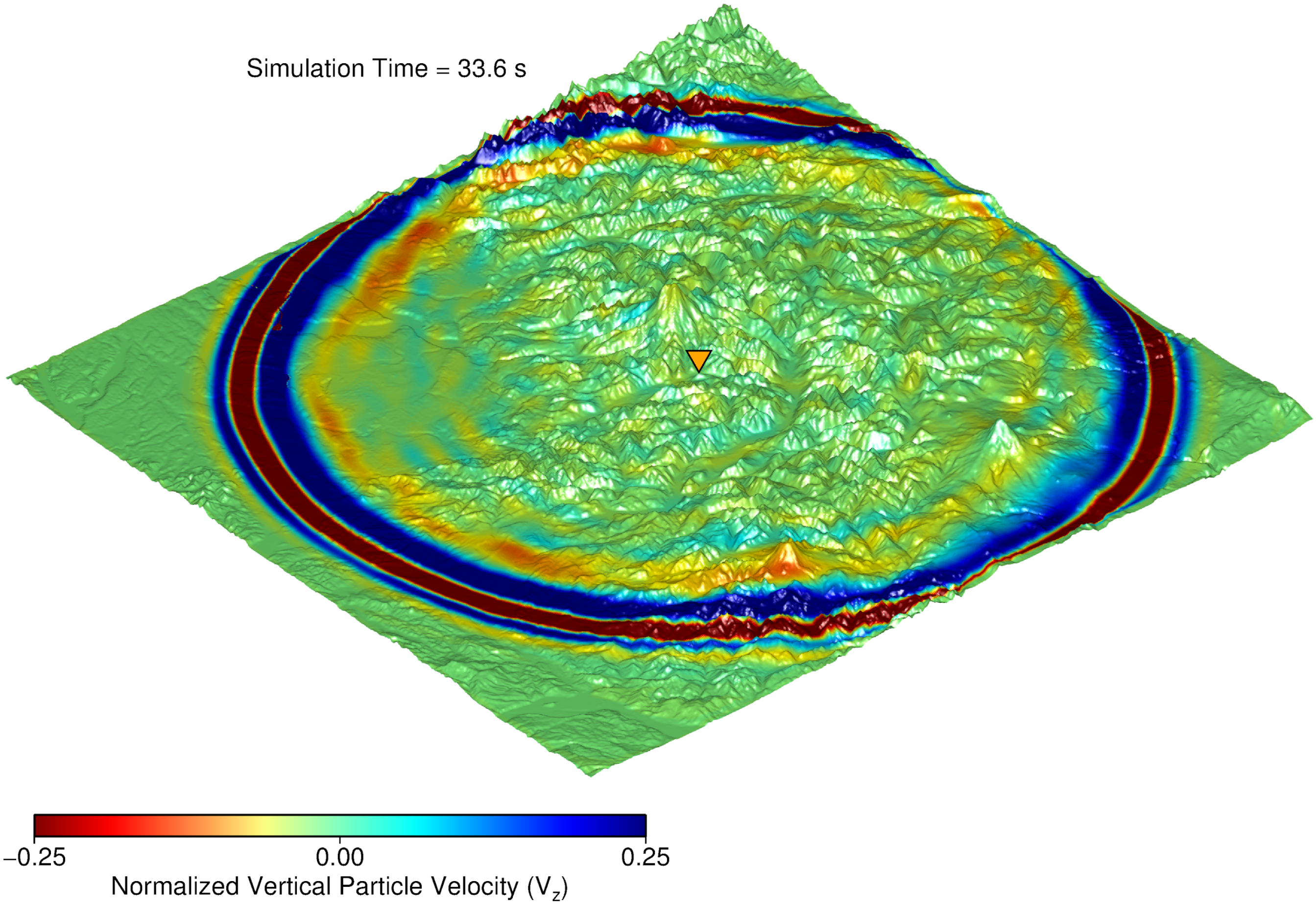

Supplemental Figure 4. Simulation snapshot. Normalized surface velocity snapshot of wave propagation for station UW.LON (orange inverted triangle), at t = 33.6 s relative to source origin time.

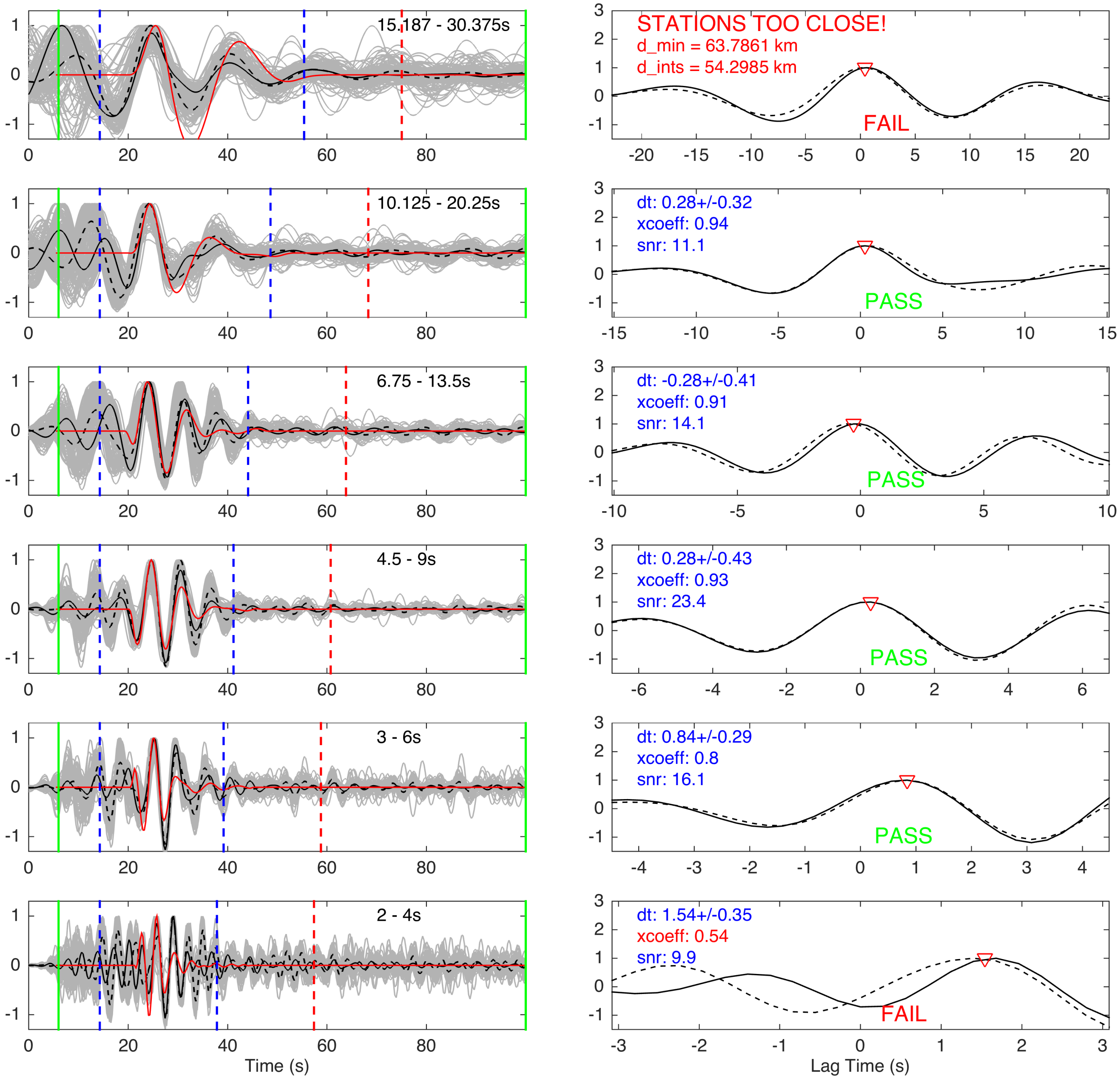

Supplemental Figure 5. Phase measurements. (left) Time delays are measured between the causal/acausal (solid/dashed) empirical Green's functions and synthetic signal (red). SNR values are calculated from period-dependent correlation windows (signal-blue sections; noise-red to second green line sections). Uncertainties in the time delay are calculated similarly, but using monthly stacks of EGFs (gray signal). **(right)** Passed time-delays are subject to several constraints including; minimum SNRs/cross-correlation coefficients, maximum time-delays, and minimum interstation distances.

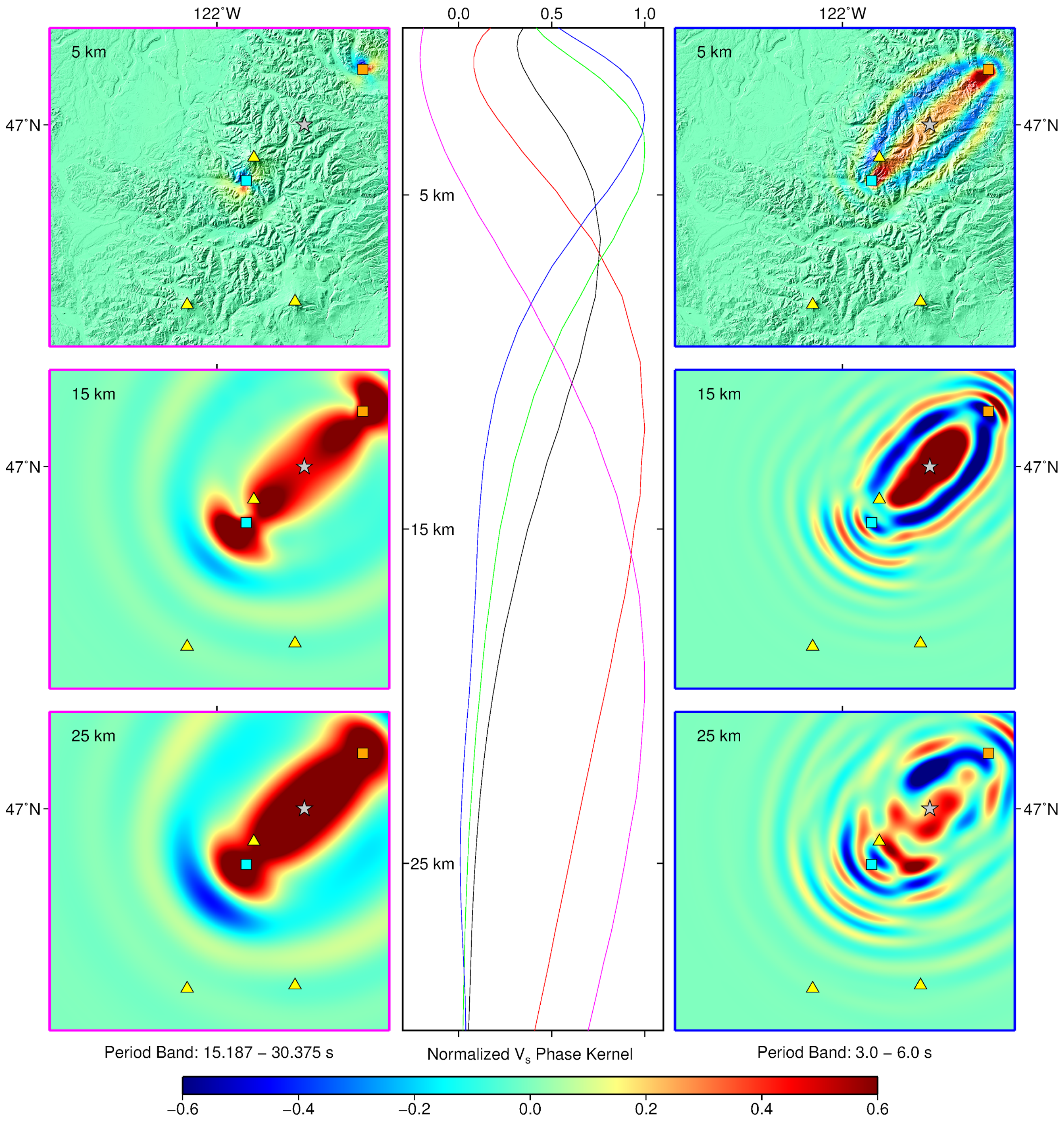

Supplemental Figure 6. Shear wave velocity phase kernels. (left/right) Sensitivity kernels for two period bands, at three depths, for station-combination UW.LON-XU.N130 (blue/orange squares). **(inset)** Depth versus sensitivity for the five passed frequency bands, at the station-station path midpoint (gray star). Shorter-periods are more sensitive to shallow structure, while long-periods have maximum sensitivity deeper within the model. Digital elevation map available from the U.S. Geological Survey.

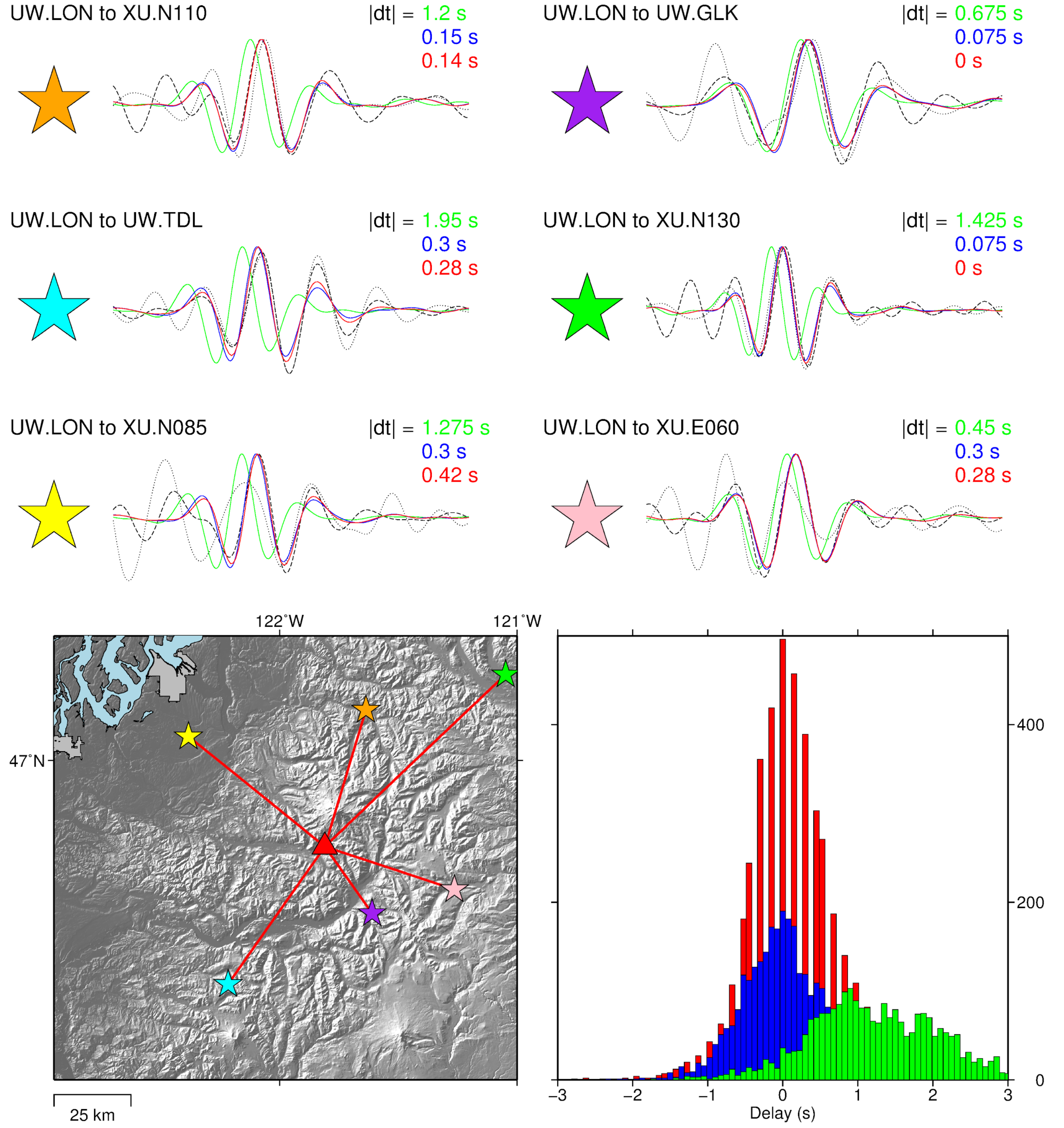

Supplemental Figure 7. Phase-delay convergence. Frequency-band dependent phase-delays converging on the synthetic signal (causal/acausal, dashed/dotted) shown at three iteration steps; iteration 1 (green), iteration 5 (blue), iteration 10 (red), for six-stations. Digital elevation map available from the U.S. Geological Survey.

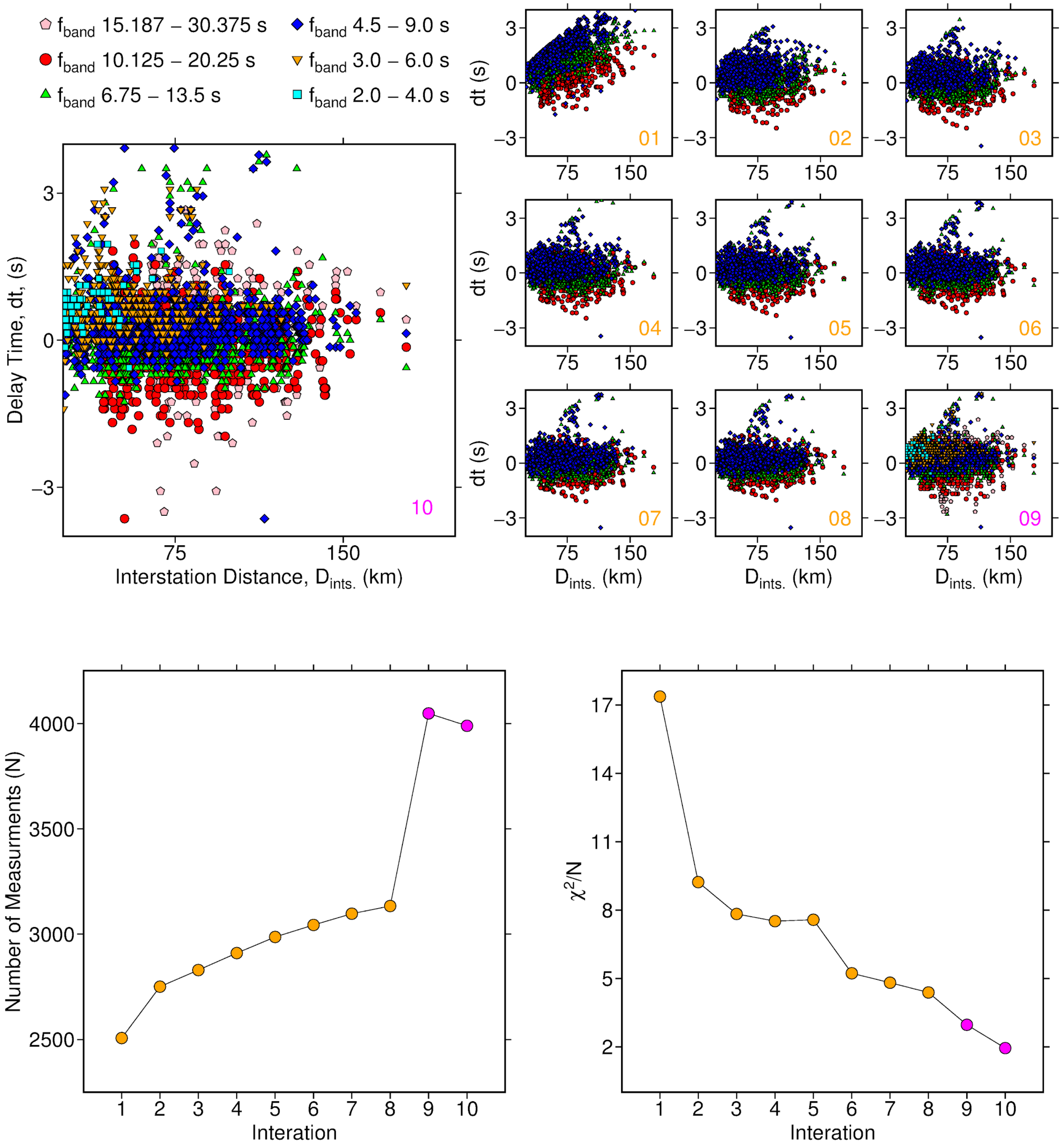

Supplemental Figure 8. Convergence statistics. **(top)** Time-delay versus interstation distance for all iteration steps and frequency bands. **(bottom-left)** The number of passed-measurements slowly increased over each iteration step as the model improved. The increase at iteration 9 is due to the addition of two higher frequency bands. **(bottom right)** Similarly, the reduced chi-squared steadily decreased.

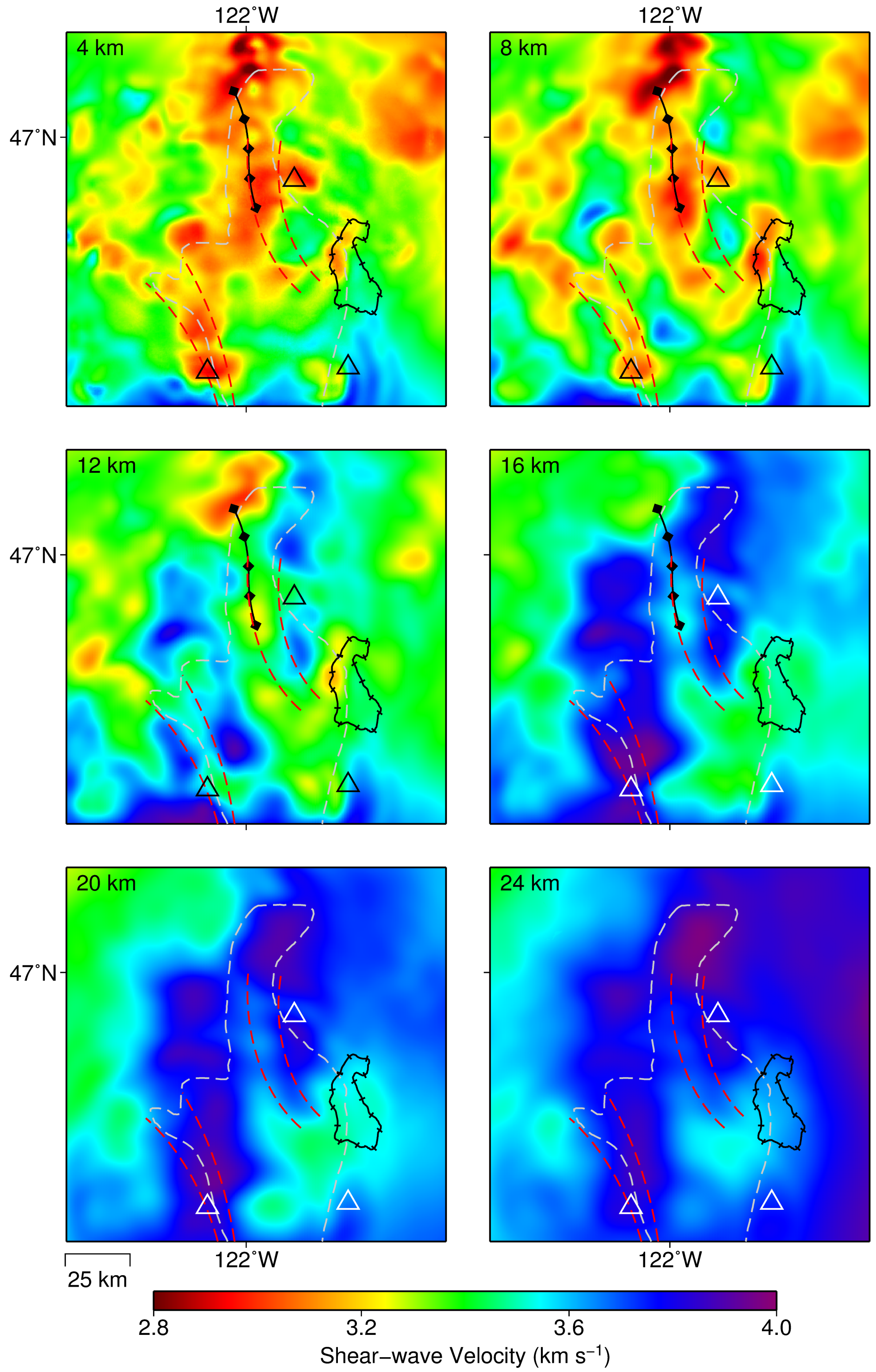

Supplemental Figure 9. Tomographic model shear-wave velocity depth slices. Constant depth slices (six) of the tomographic model. The extent of the SWCC from previous magnetotelluric studies shown as a gray-dashed line. Also shown are the Western Rainier Seismic Zone and Mount St. Helens Seismic Zone (red-dashed), the Goat Rocks Pluton (black-hachured), and nearby volcanoes (white triangles).

Resolution – Sensitivity Testing

The issue of resolution is of primary concern when addressing and validating any tomographic model. While we have shown that in the shallow crust (<10 km depth) our final model accurately correlates with previously mapped surface geology, at greater depths accuracy becomes less constrained. This reduction in accuracy with depth is a fundamental limitation of surface wave tomography, related to the frequency content used. The challenges of fully quantifying the resolution are exacerbated when dealing with an iterative full-wave tomographic workflow, such as the one used here, where the final solution slowly converges. Each iteration can be thought of as recovering a fractional portion of the total velocity perturbation, over a smoothed solution space—primarily controlled by regularization parameters within the inversion. On the other hand, the spatial resolution of the model, or the smallest resolvable feature independent of magnitude, is primarily controlled by frequency-dependent sensitivity kernels—the width of their Fresnel zones and the manner in which kernels spatially cross/overlap with one another.

We can define a test of spatial resolution, using the results of our final iteration;

1. $\mathbf{d} = \mathbf{G}\,\mathbf{m}$

where **d** is the vector of our final-iteration phase-delay measurements, **G** is the matrix of sensitivity kernels, and **m** is our final solution for velocity perturbation. We now define a new synthetic velocity-perturbation model that we will test our ability to resolve, $\mathbf{m}_S$, and generate corresponding synthetic phase-delay data, $\mathbf{d}_S$;

2. $\mathbf{d}_S = \mathbf{G}\,\mathbf{m}_s$

We test our ability to recover this synthetic velocity-perturbation model using our final-iteration sensitivity kernels and these generated synthetic phase-delay data. We use the same inversion routine (LSQR) used in our iterative tomographic process, with the same regularization parameters. Given the previous discussion on the iterative accumulation of the velocity perturbation, our resolution tests are only sensitive to the pattern of the spatial resolution recovered, and we ignore the magnitude of the recovered anomalies.

We present several resolution tests, testing the validity of the conclusions drawn from the final iterative velocity model. **Figure S10**, tests the ability to resolve two independent low-velocity anomalies, one corresponding to the Western Rainier Seismic Zone (WRSZ), the other from Mount Rainier's magma reservoir, in three separate configurations; (1) magma reservoir only, (2) WRSZ only, (3) both. **Figure S10** shows that the WRSZ and the Mount Rainier magma reservoir anomalies could not be caused by smearing from the other, e.g.; the WRSZ could not be due to a Mount Rainier magma reservoir anomaly smearing westward, nor could a Mount Rainier magma reservoir anomaly be caused by a WRSZ anomaly smearing eastward. However, **Figure S10(3)** does exhibit smearing between the two anomalies, indicating that interpretations of a connected magmatic system should not be based on tomography alone. We

reiterate that our interpretation of lateral movement of material from the WRSZ to Mount Rainier's magma reservoir is based on the geochemical signatures of assimilation of evolved crustal components, and lack of a deep sedimentary source beneath Mount Rainier, and not the apparent tomographic connection of the two systems.

Figure S11 presents our ability to constrain the depth of the Southern Washington Cascades Low Velocity Zone. (SWC-LVZ). Several different synthetic-starting models were used during testing, and the one shown best represents the observed velocity structure seen in the tomographic solution. Although the input anomaly is deeper than 25 km depth, vertical smearing extends the returned structure ~15 km upwards, although sporadically and non-coherently. While the spatial extent of the tomographic solution is robust, the SWC-LVZ as solved for in the tomographic process is likely deeper than observed in the solved velocity model. However, the SWC-LVZ volume recovered from the final tomographic model, ~12,800 km^3, is roughly equivalent to the input synthetic resolution anomaly volume ~12,566 km^3, suggesting that while depth might be only partially constrained, total size/volume is more well resolved.

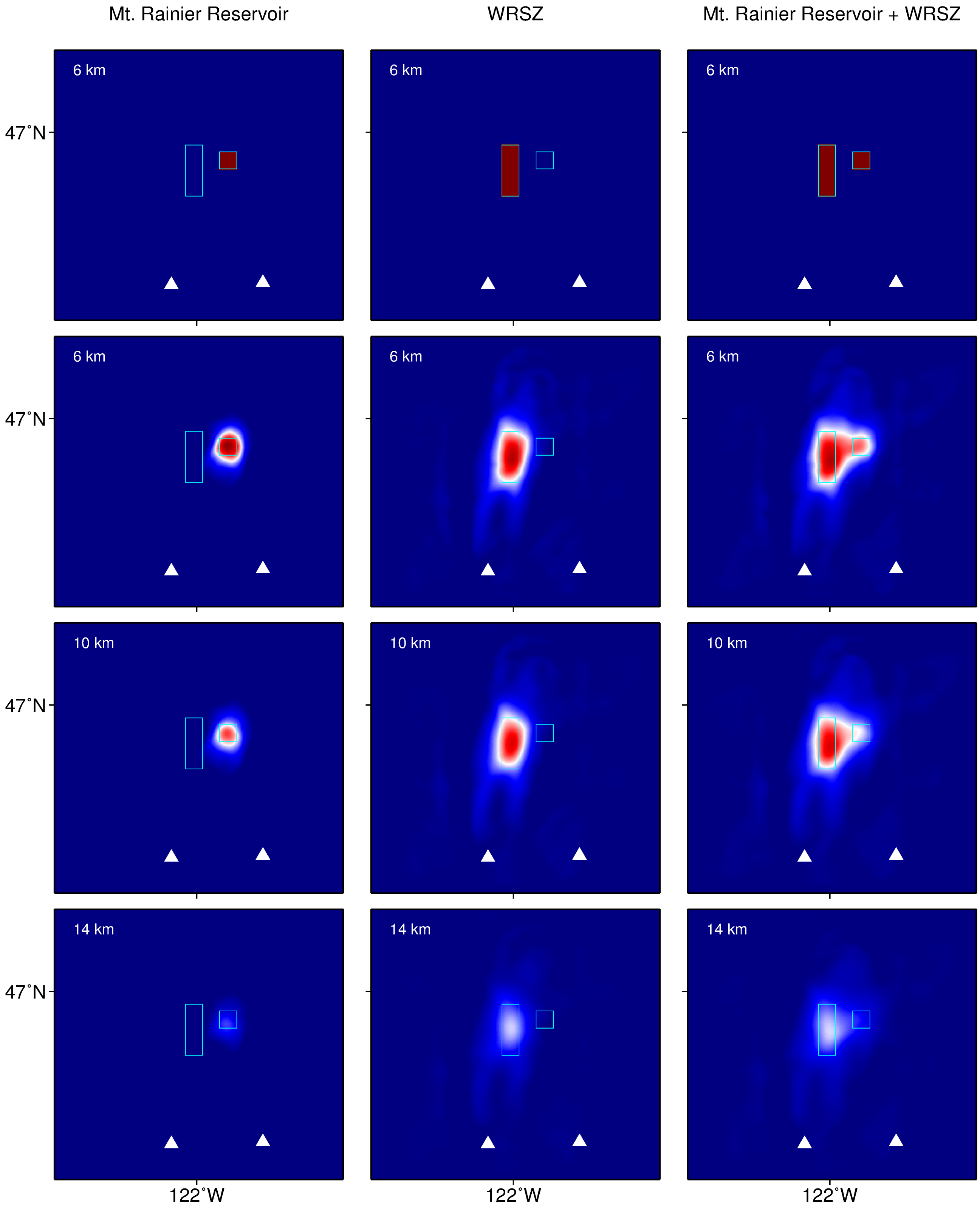

Supplemental Figure 10. Synthetic resolution tests. Synthetic tests for three cases, (1) Mount Rainier's magma reservoir only (10 x 10 km), (2) the WRSZ, and (3) both. For all three cases the synthetic anomaly extends from 2 to 10 km bsl. White triangles indicate the positions of Mounts St. Helens and Adams.

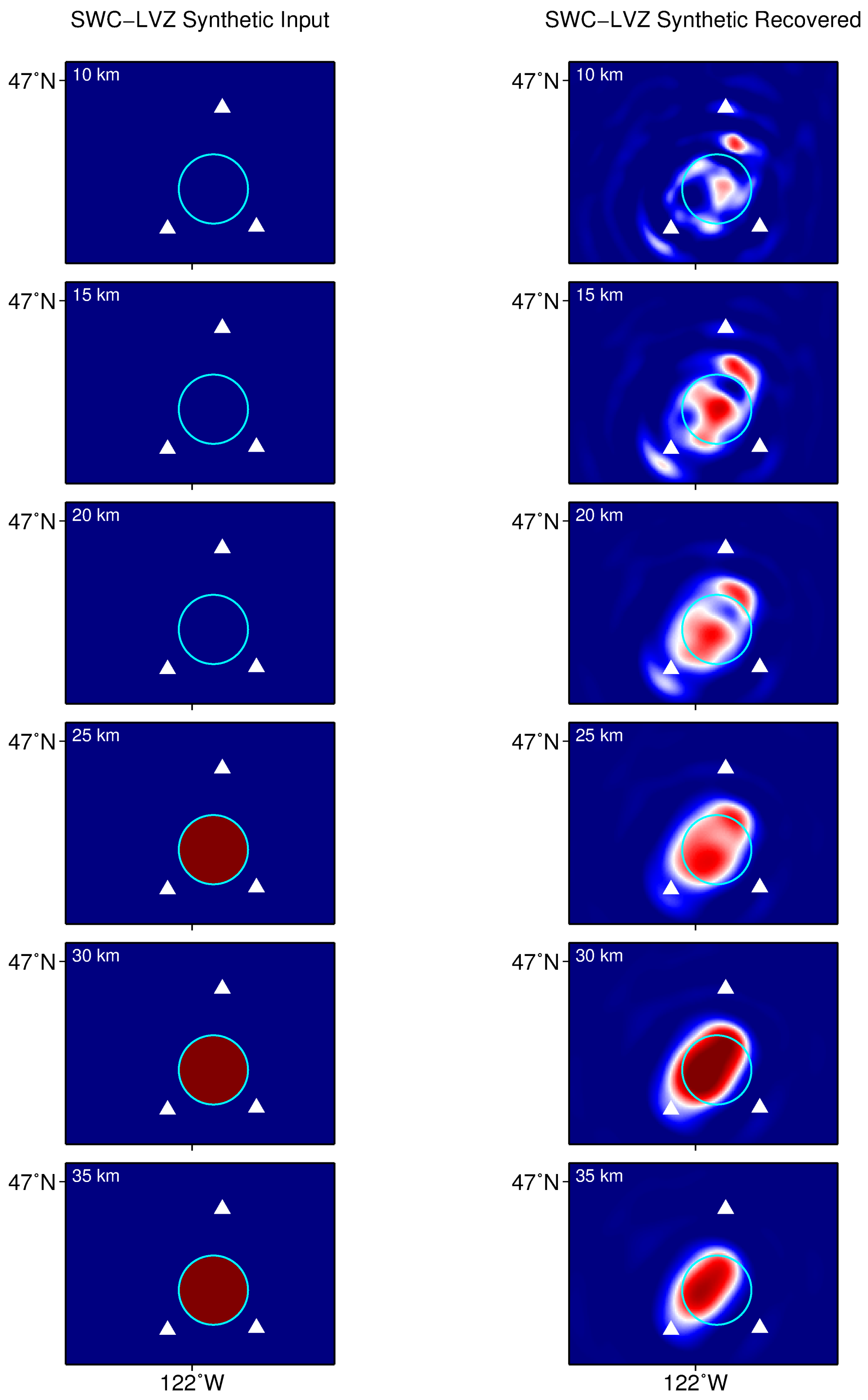

Supplemental Figure 11. Synthetic resolution test – SWC-LVZ. Resolution test for the Southern Washington Cascades Low Velocity Zone, using a cylindrical input anomaly with a 40-km diameter, extending from 25 to 35 km depth. White triangles indicate the positions of Mounts St. Helens, Adams, and Rainier.

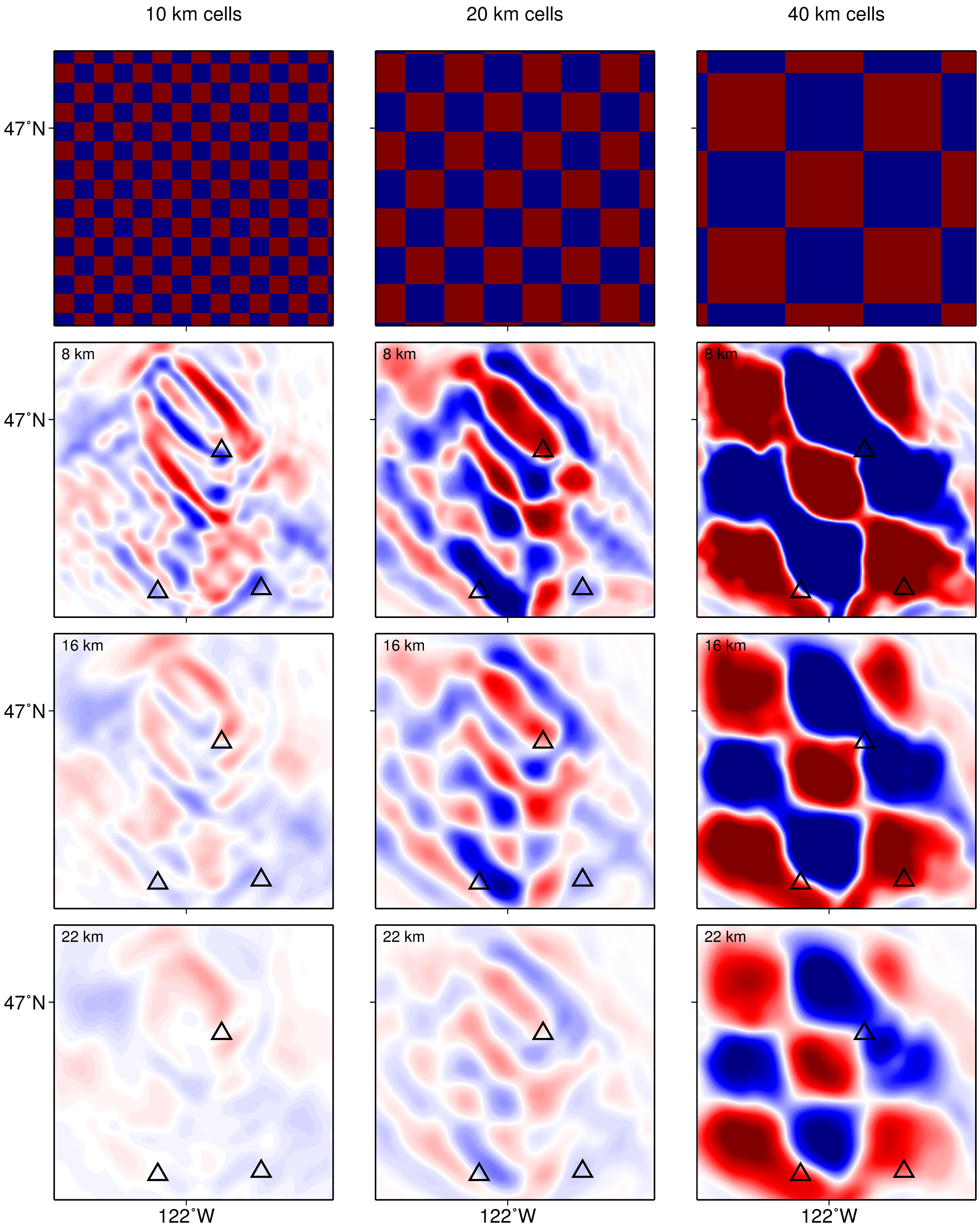

Supplemental Figure 12. Checkerboard resolution test. A resolution test for varying size constant depth checkerboards. Triangles indicate the positions of Mounts St. Helens, Adams, and Rainiers.
