# Supplementary material for: Seismic evidence for a possible deep crustal hot zone beneath Southwest Washington
Source: Sci Rep. 2017 Aug 7;7:7400. doi: 10.1038/s41598-017-07123-w (PMC5547095; doi:10.1038/s41598-017-07123-w)
Supplement: Supplementary file 2 — Tomography Results Animation Legend [file 41598_2017_7123_MOESM2_ESM.pdf]

Seismic evidence for a possible deep crustal hot zone beneath Southwest Washington

Ashton F. Flinders[1,2]*, Yang Shen[2]

[1]U.S. Geological Survey, California Volcano Observatory, Menlo Park, California, 94025
[2]University of Rhode Island, Graduate School of Oceanography, Narragansett, Rhode Island, 02882

Supplementary Video Information

The provided animation displays the shear-wave velocity results of our 3D full waveform tomographic study of Southwest Washington (USA). We show, in order; shear wave velocity, perturbations from a 1D velocity model, perturbations at a -7% threshold, and a smoothed version of these negative perturbations. Geologic interpretations of this smoothed negative perturbation are then displayed as *Eocene sediments* (green), *magma reservoirs* (red), *Western Rainier Seismic Zone* (orange), *Southern Washington Cascades-Low Velocity Zone* (yellow). Displayed digital elevation maps and aerial imagery are available from the United States Geological Survey.
